# Insights into glucosinolate accumulation and metabolic pathways in *Isatis indigotica* Fort.

**DOI:** 10.1186/s12870-022-03455-6

**Published:** 2022-02-22

**Authors:** Tianyi Zhang, Rui Liu, Jinyu Zheng, Zirong Wang, Tian’e Gao, Miaomiao Qin, Xiangyang Hu, Yuanyuan Wang, Shu Yang, Tao Li

**Affiliations:** 1 Key Laboratory of Medicinal Resources and Natural Pharmaceutical Chemistry (Shaanxi Normal University), The Ministry of Education, Xi’an, Shaanxi 710119 People’s Republic of China; 2 National Engineering Laboratory for Resources Development of Endangered Crude Drugs in Northwest China, College of Life Sciences, Shaanxi Normal University, Xi’an, Shaanxi 710119 People’s Republic of China; 3 Shaanxi Engineering Research Centre for Conservation and Utilization of Botanical Resources, Xi’an Botanical Garden of Shaanxi Province (Institute of Botany of Shaanxi Province), Xi’an, Shaanxi 710000 People’s Republic of China

## Abstract

**Background:**

Glucosinolates (GSLs) play important roles in defending against exogenous damage and regulating physiological activities in plants. However, GSL accumulation patterns and molecular regulation mechanisms are largely unknown in *Isatis indigotica* Fort.

**Results:**

Ten GSLs were identified in *I. indigotica*, and the dominant GSLs were epiprogoitrin (EPI) and indole-3-methyl GSL (I3M), followed by progoitrin (PRO) and gluconapin (GNA). The total GSL content was highest (over 20 μmol/g) in reproductive organs, lowest (less than 1.0 μmol/g) in mature organs, and medium in fresh leaves (2.6 μmol/g) and stems (1.5 μmol/g). In the seed germination process, the total GSL content decreased from 27.2 μmol/g (of seeds) to 2.7 μmol/g (on the 120th day) and then increased to 4.0 μmol/g (180th day). However, the content of indole GSL increased rapidly in the first week after germination and fluctuated between 1.13 μmol/g (28th day) and 2.82 μmol/g (150th day). Under the different elicitor treatments, the total GSL content increased significantly, ranging from 2.9-fold (mechanical damage, 3 h) to 10.7-fold (MeJA, 6 h). Moreover, 132 genes were involved in GSL metabolic pathways. Among them, no homologs of *AtCYP79F2* and *AtMAM3* were identified, leading to a distinctive GSL profile in *I. indigotica*. Furthermore, most genes involved in the GSL metabolic pathway were derived from tandem duplication, followed by dispersed duplication and segmental duplication. Purifying selection was observed, although some genes underwent relaxed selection. In addition, three tandem-arrayed *GSL-OH* genes showed different expression patterns, suggesting possible subfunctionalization during evolution.

**Conclusions:**

Ten different GSLs with their accumulation patterns and 132 genes involved in the GSL metabolic pathway were explored, which laid a foundation for the study of GSL metabolism and regulatory mechanisms in *I. indigotica*.

**Supplementary Information:**

The online version contains supplementary material available at 10.1186/s12870-022-03455-6.

## Background

*Isatis indigotica* Fort., belonging to Brassicaceae, is widely used in the food, pharmaceutical and cosmetics industries, and its dried leaves and roots, named “Da Qing Ye” and “Ban Lan Gen”, have been proven to have antiviral and antifungal effects and to activate the immune system [1, 2]. There are numerous compounds in *I. indigotica*, such as indole alkaloids, lignan metabolites, radix isatidis polysaccharides and glucosinolates. Indigo, mainly extracted from leaves, is a common blue dye extensively used in the textile industry for its safe and environmentally friendly features. Indirubin can be used as antileukemia drug [3–5]. Moreover, the seed oil of this plant has potential value as an edible oil [6].

Glucosinolates (GSLs), known as secondary metabolites in plants, are widely distributed in over 3000 species in 16 families, and are well studied in *Arabidopsis thaliana* [7]. The structure of GSLs consists of three units, a β-D-thioglucose residue, a sulfide oxime group and a variable side chain group (R-group). To date, many GSLs have been well characterized [8, 9] and are thought to be amino acid derivatives broken into three categories, namely, aliphatic, aromatic and indole. Aliphatic GSLs are derived from linear or branched-chain amino acids, including methionine, valine, leucine, isoleucine and alanine. Aromatic GSLs originate from aromatic amino acids, phenylalanine and tyrosine. The precursor amino acid of indole GSLs is tryptophan. The mustard oil bomb system is mainly made up of GSLs and myrosinases, the corresponding β-glucosidases of GSLs, which play prominent roles in plant-herbivore and plant-pathogen interaction processes [10]. Some isothiocyanates, such as sulforaphane, show good anticancer ability [11]. Brassicaceae vegetables rich in GSLs were proven to be helpful in protecting the liver and other organs [12]. Epigoitrin is the hydrolysate of epiprogoitrin (EPI), a dominant GSL in *I. indigotica,* and can be used as an antiviral compound [4, 13] and allelochemical [14]. Currently, an increasing number of researchers focus on GSLs and their functions.

The biosynthetic pathways of GSLs have been well studied in *Arabidopsis* [15, 16] and *Brassica rapa* [17]. Several amino acids, including isoleucine, methionine, phenylalanine and tyrosine, can get elongated to form homo amino acids, and the elongation route of methionine has been well studied [16]. Two branched-chain amino acid aminotransferases (BCAT4 and BCAT6) catalyze methionine and homomethionine into the corresponding 2-oxo acid in the cytoplasm and subsequently transport it into the chloroplast for a three-step cycle. First, methylthioalkylmalate synthase family members (MAMs) condense 2-oxo acid with acetyl-CoA into 2-malate derivatives. MAM1 and MAM3 prefer short- and long- chain GSLs, respectively, and MAM3 usually contributes to the elongation process. Second, isopropylmate isomerases (IPMIs) isomerize 2-malate derivatives and move hydroxyl groups to position 3. Third, oxidative decarboxylation occurs with the help of isopropylmalate dehydrogenases (IPMDHs), leaving a one-atom-elongated 2-oxo acid in the chloroplast. The cycle can be repeated up to 8 times in *Thellungiella halophila* [18], where 10-methylsulfinyldecyl GSL was detected. The subsequent transamination requires a chloroplast-localized enzyme, BCAT3, whose products are transported out of the chloroplast for core structure formation, but the corresponding transporter is still unknown [16]. For the elongation of aromatic GSLs, MAM3 was found to accept phenylalanine as a substrate, showing a possible role in aromatic GSL biosynthesis [19].

The formation of the core structure of GSLs is complex. Elongated amino acids as well as other original amino acids can be converted into the corresponding aldoximes, which can be oxidized by cytochrome P450 79 family members (CYP79) [20]. Then, CYP83 can catalyze these aldoximes into activated compounds, i.e., nitrile oxides for tryptophan derivatives and aci-nitro compounds for other derivatives. Next is a glutathione-conjunction step, and sulfur atoms are introduced to activate oximes by glutathione S-transferase family members (GSTs) [21]. γ-Glutamyl peptidase (GGP) removes γ-glutamate from the conjunct molecules, which is then converted into thiohydroximates with the help of SUPERROOT 1 (SUR1), an enzyme shared by aliphatic, indole and aromatic GSLs. The following steps are glycosylation and sulfation, charged by UGT74B1 or UGT74C1 and sulfotransferase gene family member 5 (ST5), respectively. Some basic GSLs, namely, methylthioalkyl GSL, benzyl GSL (glucotropaeolin, GTL) and indole-3-methyl GSL (I3M), are biosynthesized at the end of core structure formation.

Side chain modification is beneficial for GSL diversification. The modification of aliphatic GSLs starts from S-oxygenation by FMO_GS-OX_ gene family members, leading to the synthesis of a methylsulfinylalkyl GSL, such as glucoraphanin (4-methylsulfinylbutyl GSL). Then, alkenylation by alkenyl hydroxalkyl producing (AOP) gene family members produces alkenyl GSL (by AOP2) or hydroxyalkyl GSL (by AOP3) [22]. Other enzymes, such as GSL-OH in ecotype Cvi and GRS1 in radish, are responsible for some particular modifications [23]. On the other hand, the modification of indole GSL is found at positions 1 and/or 4, resulting in the formation of hydroxylated and methoxylated products, such as 4-methoxy-3- indolylmethyl GSL (4MOI3M) and 1-methoxy-3- indolylmethyl GSL (1MOI3M). In addition, sulfonated indole GSL was reported in *Isatis* spp. [24], indicating a sulfonation process, although the biosynthesis pathway has not been fully described. Moreover, R-hydroxylation (RHO) and S-hydroxylation (SHO) were reported to chirally hydrosylate 2-phenylethyl GSL in *Barbarea vulgaris*, which affected the structure of aromatic GSLs [25].

Intact GSLs do not show any biological activities until they break into smaller molecules. The breakdown process of GSLs was examined in *Arabidopsis* [26, 27]. To date, ten enzymes, including β-glucosidase 23 (BGLU23 or PYK10), BGLU26 (PEN2), BGLU28, BGLU30 and BGLU34–39 (also known as thioglucoside glucohydrolase 1–6, TGG 1–6) can degrade GSLs. TGG1 and TGG2, which are mainly expressed in the aboveground parts of *Arabidopsis*, function redundantly in abscisic acid-induced and methyl jasmonate-induced stomatal closure [28] and even influence physical defense barrier construction. Likewise, recent studies on a root-specific expressed myrosinase revealed that TGG4 and TGG5 played important roles in auxin biosynthesis and root growth regulation [29]. As an atypical myrosinase, PEN2 participates in pathogen defense in *Arabidopsis*. In addition, some cofactors could be involved in the GSL breakdown process with myrosinases. Nitrile-specifier proteins (NSPs), epithiospecifier proteins (ESPs) and thiocyanate-forming proteins (TFPs) can adjust GSL metabolism flow, resulting in the formation of nitriles, epithionitriles and thiocyanate rather than isothiocyanates [30]. On the other hand, myrosinase-binding and myrosinase-association proteins are available to increase the efficacy of glucosinolate breakdown and might be involved in defense against biotic stress [26, 31].

Many transcription factors can regulate GSL biosynthesis in plants. Among them, MYB28, 29, and 76, and MYB34, 51, and 122 can positively regulate aliphatic and indole GSL biosynthesis in *Arabidopsis*, respectively. MYC2, 3, 4 and 5 can directly interact with MYB proteins, showing redundant functions in response to jasmonic acid. Sulfur limitation 1 (SLIM1) can activate the breakdown process of GSLs under sulfur deficiency and downregulate MYB expression levels [32]. In addition, Dof1.1 and IQ-domain 1 (IQD1) can regulate the expression of GSL metabolism-related genes [33, 34]. Recent studies also verified the functions of *CAMTA3*, *CCA1*, *FRS7*, *FRS12* and *HY5* [35, 36], and an epistatic regulation network is still being constructed, indicating the complicated relationships of the transcription factors in the GSL metabolic pathway [37].

Different developmental stages, organs and tissues, as well as different treatments, have different effects on the accumulation and metabolism of GSLs. For example, the seeds and roots of Brassicaceae plants accumulated more GSLs than other organs [38]. Methyl jasmonate (MeJA) and salicylic acid (SA) had distinct impacts on GSL accumulation [39]. The distributions of GSLs were summarized in more than 130 genera [7], showing GSL variations across evolution. GSL profiles have been investigated in *Arabidopsis* [11], and GSL accumulations were also discussed in *B. rapa* [40, 41], *B. oleracea* [42], *Raphanus sativus* [43], *Bunias erucago* [44], *Isatis* spp. [24] and recently in *Erysimum* spp. [45] and *Lepidium graminifolium* [46]. However, little is known about GSL accumulation and regulation in *I. indigotica* [47–50]. Here, the GSL contents at different developmental stages, in different organs and under different treatments were determined, and the related genes were also explored and analyzed in this species.

## Results

### Glucosinolate determination in *I. indigotica*

The GSLs were investigated by LC-MS/MS on their corresponding desulfo counterparts, considering the results of previous reports [47, 51]. All the detected GSLs are listed in Table 1, Table S1 and Figs. S1, S2 and S3. Ten GSLs were identified in *I. indigotica*, and the dominant GSLs were EPI and I3M, followed by progoitrin (PRO) and gluconapin (GNA). In addition, it was speculated that six new indole GSLs could exist; these compounds showed typical GSL characteristics on LC-MS/MS spectra, i.e., sulfide oxime moiety (*m/z* = 75), neutral loss (198 Da instead of 162 Da, because of chloride ion contamination), fragments from desulfurized glycoside aglycone (*m/z =* 144, 146 and 160) and an even-numbered relative molecular mass (*m/z* = 384 or 400) (Fig. S4, [52–55])*,* but further evidence is still needed.

**Table 1:** Detected GSLs in *I. indigotica*

No.	Name	Relative molecular weight	Mass after desulphonation (*m*/*z*)	Retention time (min)	Correction coefficient	Type
1	Progoitrin	389	309	5.3	1.09	aliphatic
2	Epiprogoitrin	389	309	5.8	1.09	aliphatic
3	Sinigrin (Internal standard)	359	279	6.8	1	aliphatic
4	Gluconapin	373	293	10.2	1.11	aliphatic
5	4-hydroxy- 3-indolylmethyl GSL	464	384	11.9	0.28	indole
6	Glucobrassicanapin	387	307	13.7	0.25	aliphatic
7	Glucotropaeolin	409	329	14.7	0.95	aromatic
8	Indolyl-3-methyl	448	368	16.8	0.29	indole
9	4-methoxy- 3-indolylmethyl GSL	478	398	18.8	0.25	indole
10	(R,S)-Glucoisatisin	562	482	20.9(R) 21.1(S)	1	aliphatic
11	1-methoxy- 3-indolylmethyl GSL	478	398	23.8	0.2	indole

Notes: The retention time is chosen to sequence GSLs in Table 1. All GSLs are grouped into three types, namely aliphatic, aromatic and indole, according to their side chain structures. Correction coefficient is used to eliminate the error in content determination

### Glucosinolate content changes in different developmental periods in *I. indigotica*

**Fig. 1:**
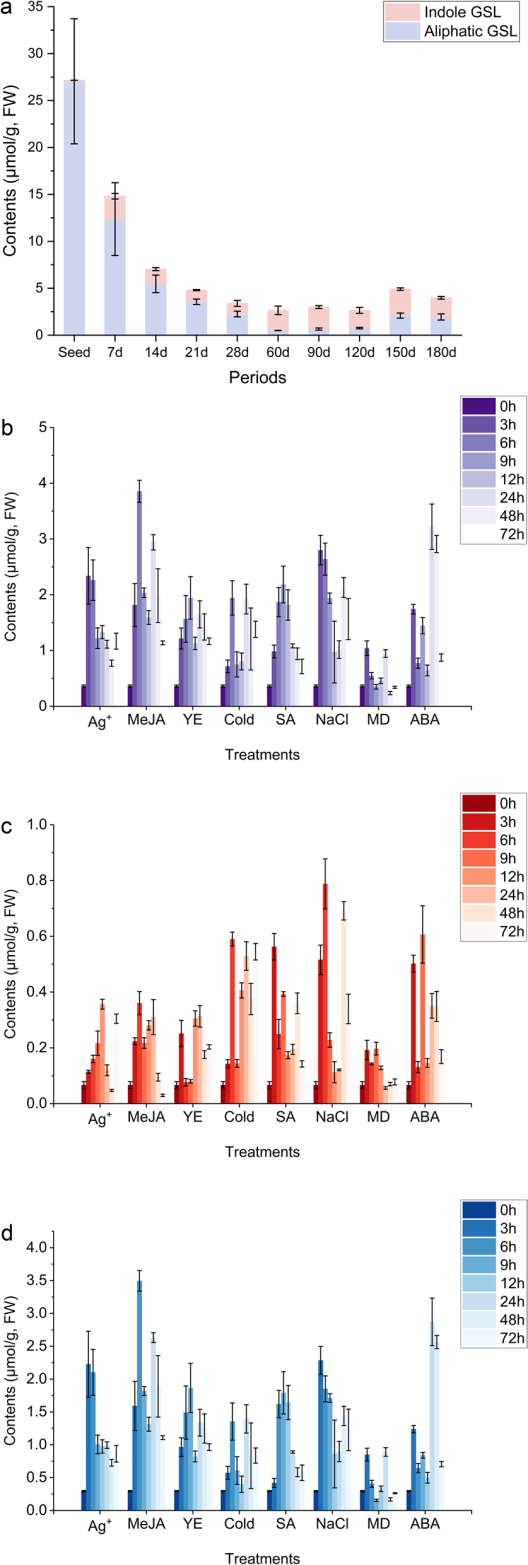
GSL contents in different developmental periods and responding to the different elicitors. Ag^+^: 10 mM silver nitrate solution; MeJA: 500 μM methyl jasmonate solution; YE: 10 g/L yeast extraction solution; Cold: 4 °C treatment; SA: 300 μM salicylic acid solution; NaCl: 0.1 mol/L sodium chloride solution; MD: mechanical damage treatment; ABA: 1 mM abscisic acid solution; **a** GSL contents in different developmental periods (seeds, 7, 14, 21, 28, 60, 90, 120, 150 and 180 DAG); Red columns show the indole GSL contents, while blue columns represent the aliphatic GSL contents; **b** Total GSL contents variations after different elicitor treatments over time (3, 6, 9, 12, 24, 48 and 72 h); **c** Aliphatic GSL content variations after different elicitor treatments over time; **d** Indole GSL content variations after different elicitor treatments over time; Mean ± SD values (*n* = 3) were shown in each column. The detailed results are listed in Table S2

The GSL accumulation patterns during the different developmental periods were measured (Fig. 1a, detailed in Table S2b). The total GSL content was the highest (27.2 μmol/g, FW) in seeds (Fig. 1a). After germination, the total GSL content decreased sharply until 60 DAG (days after germination) and then increased gradually, with aliphatic GSLs being the main compound. In particular, limited indole GSLs (0.10 μmol/g) were detected in seeds, while aliphatic GSLs contributed to more than 99% of the total GSL content. In seedlings, indole GSLs remained relatively stable. The levels of some specific GSLs, such as PRO, EPI and GNA, apparently decreased from germination to 60 DAG (Table S2b). In addition, R-glucoisatisin and S-glucoisatisin (combined as glucoisatisin, GIT) were detected before 28 DAG, with a peak value (0.98 μmol/g) at 7 DAG. Moreover, indole GSL distribution patterns were also observed, and I3M was not detected in seeds. The indole GSL contents fluctuated between 1.1 μmol/g (28 DAG) and 2.8 μmol/g (150 DAG). Three indole GSLs with side-chain modifications, namely, 4-hydroxy-3-indolylmethyl GSL (4OHI3M), 4MOI3M and 1MOI3M, reached the highest values at 60 (0.12 μmol/g), 120 (0.38 μmol/g) and 150 (0.93 μmol/g) DAG, respectively. Interestingly, 1MOI3M showed a similar variable pattern to aliphatic GSLs, and the lowest value (0.24 μmol/g) appeared at 60 DAG.

### Glucosinolate content changes in different organs in *I. indigotica*

The GSL contents in ten organs were examined, as shown in Table 2. Reproductive organs were more enriched for GSLs than vegetative organs, followed by roots and the remaining aerial parts. Few GSLs distributed in mature stems and leaves. Aliphatic GSLs were dominant in all organs, accounting for more than 70% of the total GSLs. More GSLs were distributed in fresh leaves and stems than in senescent organs. The most abundant GSLs were EPI (from 0.34 to 13.16 μmol/g) and PRO (from 0.04 to 8.87 μmol/g), as shown in Table S2c. Interestingly, glucotropaeolin (GTL) and glucobrassicanapin were only detected in reproductive organs, and glucobrassicanapin could barely be detected by LC-MS/MS. Additionally, PRO, EPI and GNA were the three dominant GSLs in the early reproductive growth period, reaching 95% of the total GSLs and sharing similar distribution patterns. However, the distribution patterns of the other GSLs were diverse. I3M was more abundant in roots, flowers and fruits but less abundant in stems and leaves, and 1MOI3M was mainly distributed in roots (over 1.5 μmol/g).

**Table 2:** Glucosinolate contents in different organs in *I. indigotica*

Organs	Total GSLs (μmol/g, FW)	Aliphatic GSLs (μmol/g, FW)	Percentage (%)	Indole GSLs (μmol/g, FW)	Percentage (%)	Aromatic GSLs (μmol/g, FW)	Percentage (%)
Main roots	10.257 ± 1.202^b^	7.418 ± 0.890^b^	72.3	2.839 ± 0.312^c^	27.7	N.D.	–
Lateral roots	18.053 ± 1.648^c^	15.015 ± 0.857^c^	83.2	3.039 ± 0.790^c^	16.8	N.D.	–
Mature stems	0.392 ± 0.038^a^	0.365 ± 0.032^a^	93.2	0.027 ± 0.0059^a^	6.8	N.D.	–
Middle stems	0.736 ± 0.039^a^	0.732 ± 0.039^a^	99.4	0.005 ± 0.001^a^	0.6	N.D.	–
Fresh stems	1.505 ± 0.068^a^	1.490 ± 0.066^a^	99.0	0.016 ± 0.001^a^	1.0	N.D.	–
Mature eaves	0.429 ± 0.057^a^	0.405 ± 0.056^a^	94.5	0.024 ± 0.002^a^	5.5	N.D.	–
Fresh leaves	2.595 ± 0.215^a^	2.556 ± 0.211^a^	98.5	0.038 ± 0.004^a^	1.5	N.D.	–
Buds	20.629 ± 1.746^c^	20.540 ± 1.727^c^	99.6	0.073 ± 0.014^a^	0.3	0.016 ± 0.004^a^	0.1
Flowers	22.791 ± 0.837^c^	22.124 ± 0.699^c^	97.1	0.488 ± 0. 084^ab^	2.1	0.179 ± 0.054^b^	0.8
Immature fruits	21.352 ± 1.437^c^	20.254 ± 1.297^c^	94.9	0.864 ± 0.090^b^	4.0	0.234 ± 0.050^b^	1.1

Note: The data show the GSL contents in different organs. Discrepant lowercase letters represent different significant levels. All data are displayed with means ± SD values (*n* = 3). N.D. means “not detected”

### Glucosinolate content changes under different treatments in *I. indigotica*

The effects of eight elicitors on GSL accumulation were investigated (Fig. 1b-d, Table S2d). The results revealed that MeJA, NaCl and ABA had the most remarkable effects on the total GSL accumulations, and the peak contents were 3.85 (10.7-fold compared to control groups, 6 h), 2.96 (7.7-fold, 3 h) and 3.32 μmol/g (8.9-fold, 24 h), respectively. However, mechanical damage did not have a significant influence, with only a 2.6-fold change at the peak (3 h). For aliphatic GSLs, SA, low temperature, NaCl and ABA resulted in 8.4-fold (3 h), 8.8-fold (6 h), 11.7-fold (6 h) and 9.1-fold (9 h) increases, respectively. Notably, SA, NaCl and ABA treatments had clear effects on PRO and EPI, and the peak times were 3 (8-fold), 6 (10-fold) and 9 h (11-fold), respectively. Furthermore, the indole GSL contents increased after AgNO_3_, MeJA, NaCl and ABA treatments, and the peak values reached 2.23 μmol/g (3 h), 3.49 μmol/g (6 h), 2.28 μmol/g (3 h) and 2.87 μmol/g (24 h), respectively. The different elicitors had different impacts on I3M, one of the main GSLs, and its contents reached their highest values (3.01 μmol/g and 2.67 μmol/g) after 6 h of MeJA and 24 h of ABA treatments. In contrast, AgNO_3_ and NaCl had obvious impacts on I3M, with peak values (1.84 and 1.89 μmol/g) found after 3 h. For SA and YE treatment, the contents of I3M reached 1.51 μmol/g and 1.65 μmol/g over 9 h.

### Exploration of Glucosinolate metabolic pathways in *I. indigotica*

Based on the genome database of *I. indigotica* from our lab (the raw data in the NCBI database can be accessed with accession number PRJNA612129), the genes involved in GSL biosynthesis and breakdown pathways were explored (Table S3). There were 132 genes involved in the GSL metabolic process (Table 3), of which 70 genes were related to the biosynthesis process, 38 genes played roles in the breakdown process, 2 genes worked as transporters, and 22 genes regulated gene expression as transcription factors. In addition, there were 32 homologous chromosome segments with base deletions and insertions, perhaps due to nonfunctionalization.

**Table 3:** Number of glucosinolate metabolic genes

Categories	Process	Number of genes	Number of similar sequences	Total
Biosynthesis	Side-chain elongation (SE)	14	2	16
	Core structure formation (CF)	26	4	30
	Side-chain modification (CM)	19	3	22
	Co-substrate pathways (CS)	11	2	13
Breakdown	Myrosinase (MY)	21	10	31
	Co-factor involved in glucosinolate breakdown (CB)	17	11	28
Transcription factor		22		22
Transportation		2		2
Total		132	32	164

**Fig. 2:**
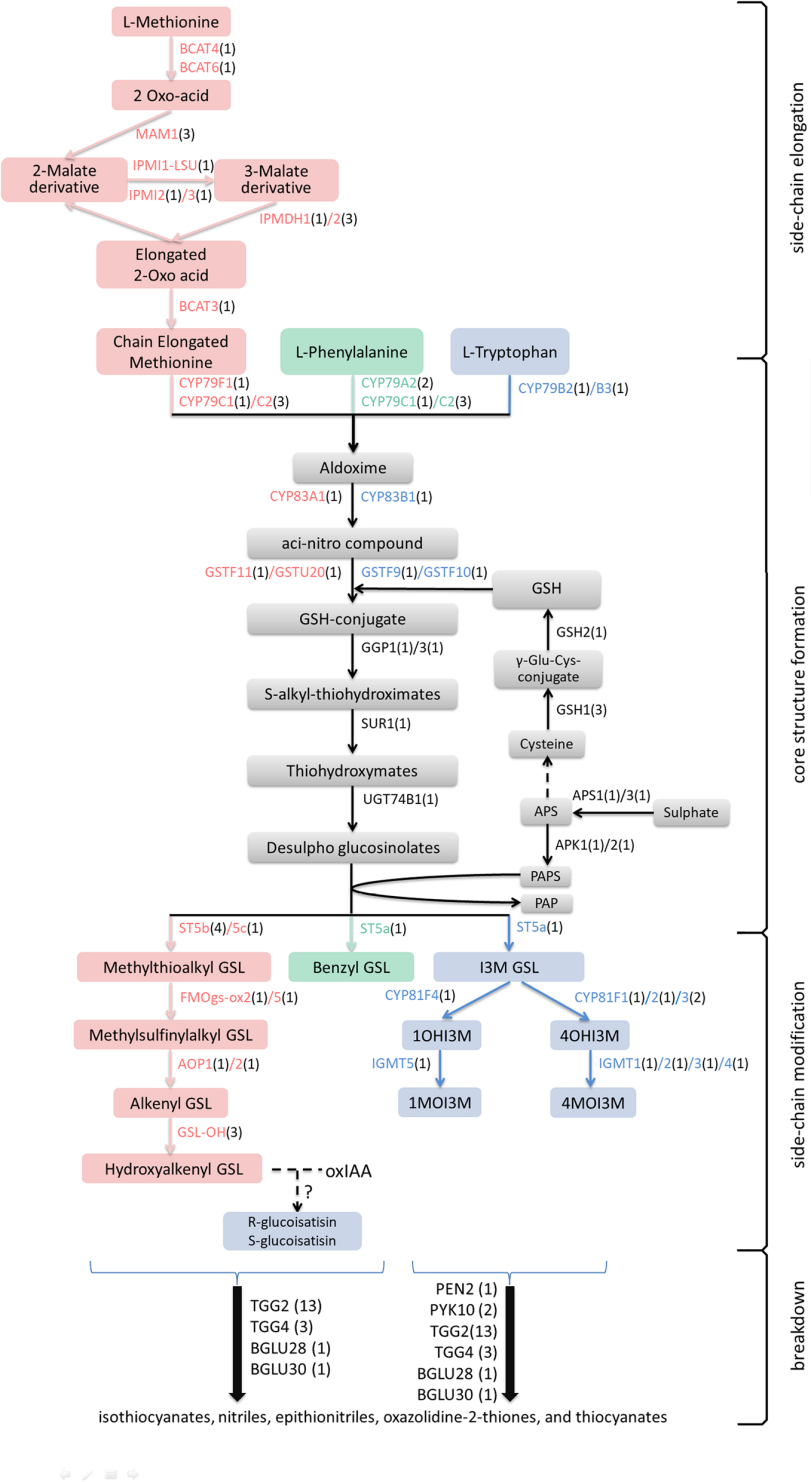
The GSL metabolic pathway of *I. indigotica.* Numbers in brackets represent the numbers of genes homologous with *Arabidopsis*; Red, green and blue words or squares represent aliphatic, aromatic and indole GSL metabolism-related genes or products, respectively; Dashed lines indicate the predicted reaction or multiple-step reactions; BCAT: branched-chain amino acid aminotransferase; MAM: methylthioalkylmalate synthase; IPMI: isopropylmalate isomerase; IPMDH: isopropylmalate dehydrogenase; CYP: cytochrome P450 monooxygenase; GST: glutathione S-transferase; SUR: S-alkyl-thiohydroximate lyase; UGT: uridine 5′-diphospho-glucuronosyltransferase; ST: sulphotransferase; FMO: flavin-containing monooxygenase; AOP: alkenyl hydroxyalkyl producing; IGMT: indole glucosinolate O-methyltransferase; TGG: thioglucoside glucohydrolase; PEN2: penetration-resistance gene 2; BGLU: beta glucosidase; APS: adenosine 5′-phosphosulphate; APS: adenosine 5′-phosphosulphate; APK: APS kinase; GSH: glutathione; PAPS: 3′-phospho-adenosine-5′-phosphosulphate; PAP: 3′-phospho-adenosine 5′-phosphate

**Fig. 3:**
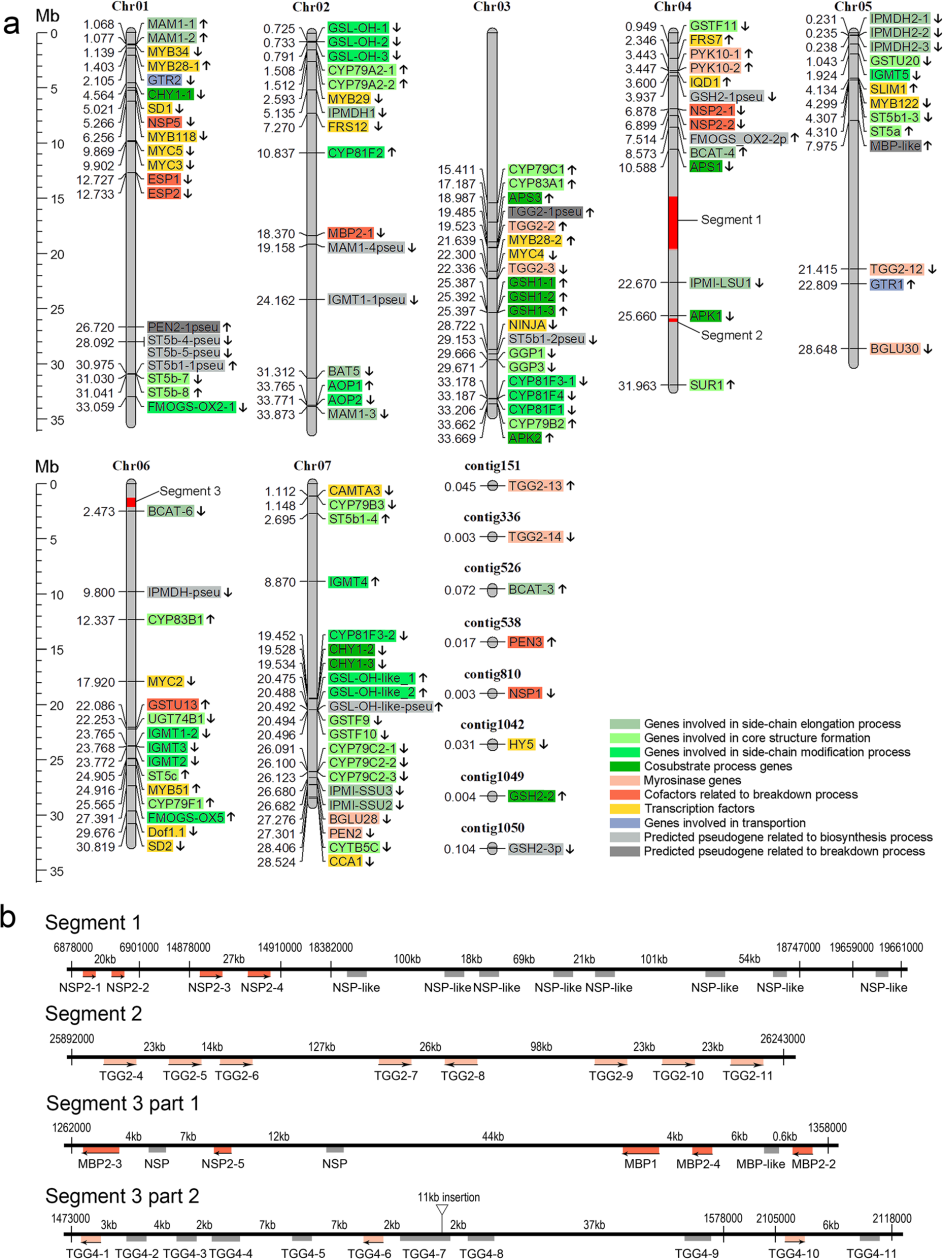
Chromosome locations of GSL metabolism genes. **a** Numbers on the left show the location (Mb) of genes on pseudochromosomes; Arrows on the right are the relative direction; Genes with gradient green rectangle background represent side-chain elongation process, core structure formation process, side-chain modification process and co-substrate process, respectively; Light red rectangles represent myrosinases, and dark red shapes represent co-factor; Yellow rectangles mean transcription factors; Similar sequences of biosynthetic genes and breakdown genes are filled with light and dark grey, respectively; Boxes of transportation genes are painted with blue; Segments filled in orange on the pseudochromosome are enlarged in **b**. Chr: pseudochromosome. **b** The linear distributions of GSL breakdown genes on Chr04 and Chr06. Light or dark red squares represent genes with complete ORF; Grey squares mean nonfunctionalization fragments; the distance is shown above lines

Based on the GSL metabolic pathways of *Arabidopsis* and those from other studies [16, 56, 57], the GSL metabolic pathway of *I. indigotica* is shown in Fig. 2. Sixty-eight genes, including core enzyme genes (*CYP79* and *CYP83*), were single-copy genes, while 17 enzymes had two or more functional copies. In particular, 13 functional genes were homologous to *AtTGG1* (*AT5G26000*) or *AtTGG2* (*AT5G25980*), and that number was greater than that in other Brassicaceae plants (Table S4). As shown in Fig. 3a, the GSL pathway genes were distributed on all seven pseudochromosomes, revealing a certain concentrated distribution, with no additional clustering. One hypothesis is that the GSL metabolic pathway evolved step by step, and gene recruitment did not depend on proximity. Up to 33 genes were located on Chr04 and Chr06, while 13 genes were located on Chr05. There were 19 pairs (45 genes, Fig. 3) of tandem duplicates, more than any other repeat type (Table 4), implying the importance of tandem repeat events [58]. The genes involved in the GSL breakdown process are shown in Fig. 3b. There were three prominent regions where GSL-related genes were densely distributed, namely, NSP-like loci, TGG-like loci and MBP-like loci, which were located near each other on Chr04 or Chr06. However, there were some sequences that seemed nonfunctionalized due to base deletions or insertions. In contrast, 5 NSP loci, 5 myrosinase-binding protein (MBP) loci and 11 TGG loci were relatively complete.

**Table 4:** Duplication type and the corresponding number of glucosinolate metabolic genes

Duplication type	Gene numbers in GSLs metabolic pathway	Gene numbers around the whole genome
Singleton	0	3224
Dispersed	37	19,317
Proximal	17	1770
Tandem	45	3242
WGD or segmental	33	7226
Total	132	34,779

**Fig. 4:**
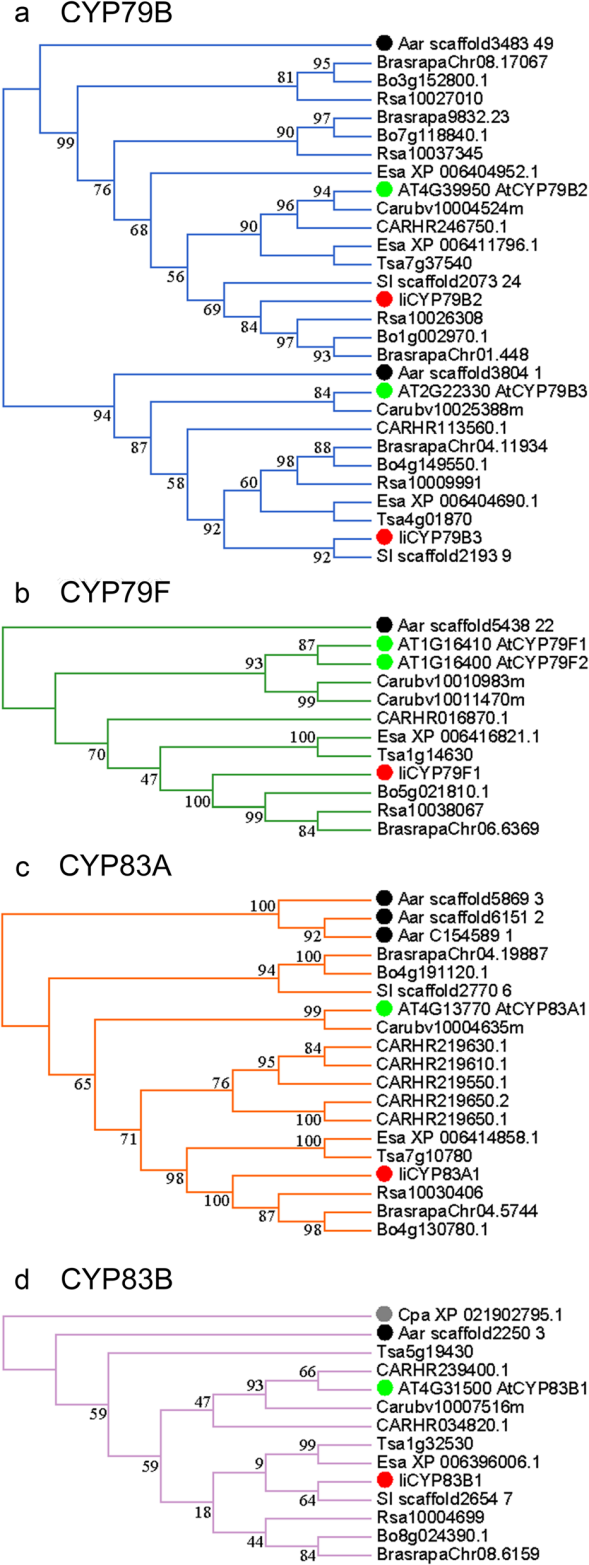
Phylogenetic trees of GSL metabolic pathway core genes. **a** CYP79B; **b** CYP79F; **c** CYP83A; **d** CYP83B; Different species are distinguished by different colours, green for *Arabidopsis*, red for *I. indigotica*, black for *Aethionema arabicum* (the basal species of Brassicaceae), and grey for *C. papaya* (a closely related species of Brassicaceae). All phylogenetic trees are constructed by FastTree 2.1 using 1000 bootstrap replicates. Detailed information can be found in Fig. S6 and Table S5

The cytochrome P450 family (CYP), 2-oxoglutarate-dependent dioxygenase family (2OGD) and MAM genes played important roles in the GSL metabolic pathway, and phylogenetic trees were constructed for *I. indigotica* and other Brassicaceae species, with *Carica papaya* and *Moringa oleifera* as the outgroup species (Fig. 4, Figs. S5, S6 and S7 and Table S5). In terms of GSL profiles [7, 8] and the relevant core genes (Fig. 4, Table S5, Figs. S5, S6 and S7), our study results supported that *I. indigotica* has a close relationship to *Sisymbrium irio*, *Brassica* spp. and *R. sativus*. Different GSL profiles existed in different plants. *I. indigotica* mainly accumulated hydroxyalkenyl GSLs, while *Arabidopsis* tended to accumulate methylsulfinylalkyl GSLs. Moreover, some genes were absent in *I. indigotica*, including *MAM2*, *MAM3*, *CYP79F2*, *UGT74C1*, *FMO*_*GS-OX*_*1/3/4/6/7*, *AOP3*, *BZO1p1*, *MYB76*, *MYB115*, *NSP3* and *NSP4*, based on the genomic data (Table S6, Fig. S8). Among them, *MAM3* and *CYP79F2* participate in long-chain aliphatic GSL biosynthesis, while *AOP3* catalyzes the transition of methylsulfinylalkyl GSLs to hydroxyalkyl GSLs. Furthermore, FMO_GS-OX_ enzymes could also result in the absence of long-chain aliphatic and hydroxyalkyl GSLs [59].

### Selection on genes involved in Glucosinolate metabolism in *I. indigotica*

**Fig. 5:**
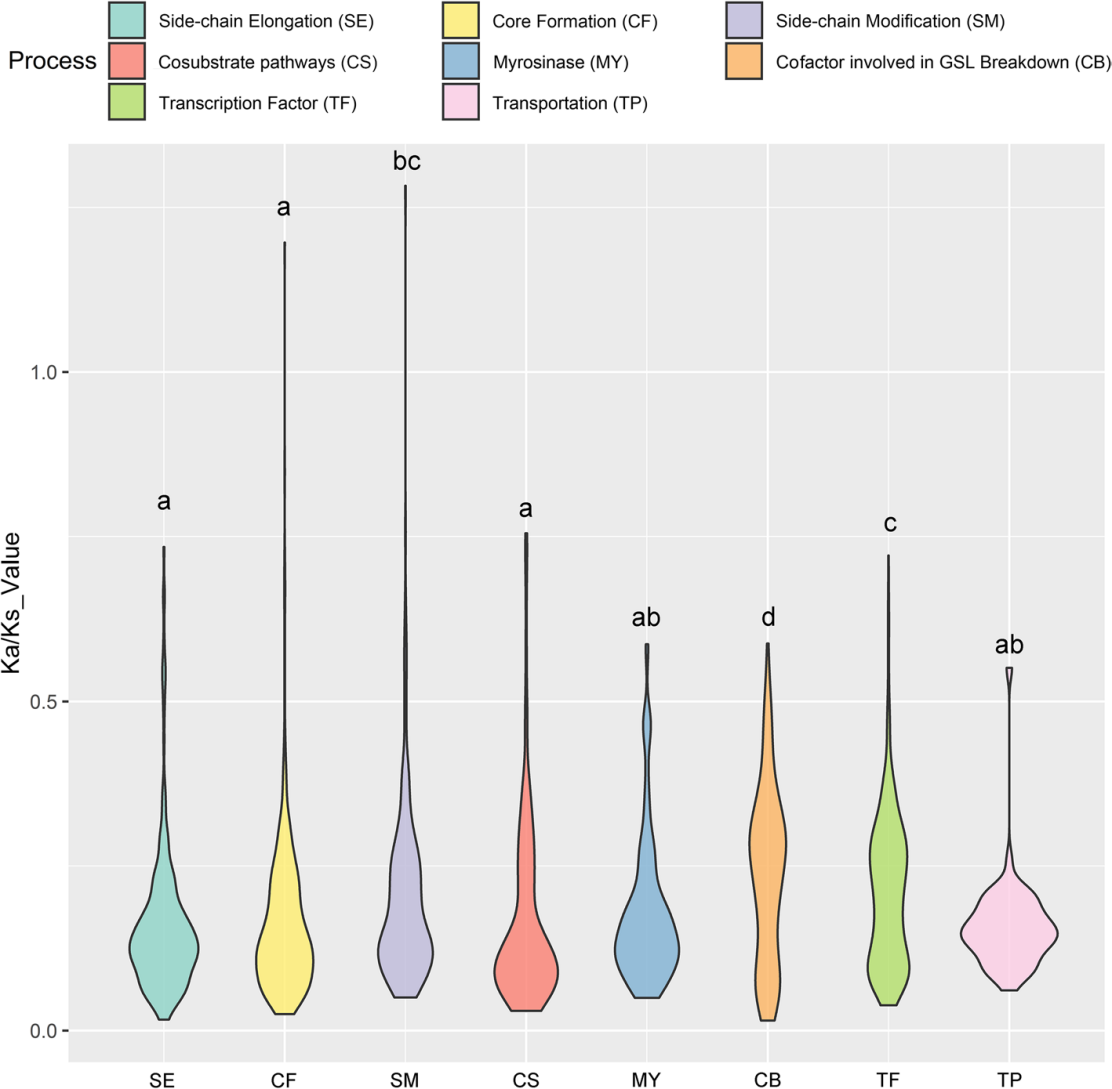
The Ka/Ks ratio distribution of homolog gene pairs from eight different processes. Abbreviations behind X-axis represent different processes in GSL metabolic pathway: SE for side-chain elongatio, CF for core structure formation, SM for side-chain modification, CS for co-substrate pathway, MY for myrosinase, CB for co-factor involved in GSL breakdown, TF for transcription factor and TP for transportation. Lowercase letters above each column are subset divisions after multiple comparisons (Kruskal-Wallis H test with Bonferroni significance level correction). Genes undergo a purifying selection on the whole, though some gene pairs have a higher ratio than 0.5 (weak positive selection). Gene pairs can be found in Table S6. The group division and the ratio details are listed in Table S7

Selection always affects gene evolution in plants. Using the proteins encoded by glucosinolate-related genes in *I. indigotica* as references, we searched the protein database of 25 other Brassicaceae species by BLASTp, and vice versa. Bidirectional best hits were regarded as homologous genes and used for further analysis (Fig. S6, Table S8). The ParaAT workflow [60] was carried out to calculate nonsynonymous nucleotide substitution rates (Ka), synonymous nucleotide substitution rates (Ks) and their ratios (Ka/Ks) for gene pairs. The results are shown in Table S7. The GSL pathway was divided into eight groups (Table S3), i.e., side-chain elongation (SE), core structure formation (CF), side-chain modification (SM), cosubstrate pathways (CS), myrosinase (MY), cofactors involved in glucosinolate breakdown (CB), transcription factors (TF) and transportation (TP). As illustrated in Fig. 5, most genes were under selective pressure during evolution. Interestingly, SM, CB and TF processes underwent more relaxed selection than SE, CF and CS processes. Additionally, CF, SM and MY each were divided into subgroups. For CF (Fig. S9a), no significant differences were observed among key enzymes (CYP79 and CYP83) shared between aliphatic and indole GSL biosynthesis (GGP, SUR and UGT) and their respective biosynthetic enzymes (GST and ST5). Nevertheless, a discrepancy in selection pressure was found between the atypical and typical myrosinases (Fig. S9c), as well as between genes involved in aliphatic and indole GSL modifications (Fig. S9b).

### Analysis of the key side-chain modification genes related to *GSL-OH* in *I. indigotica*

**Fig. 6:**
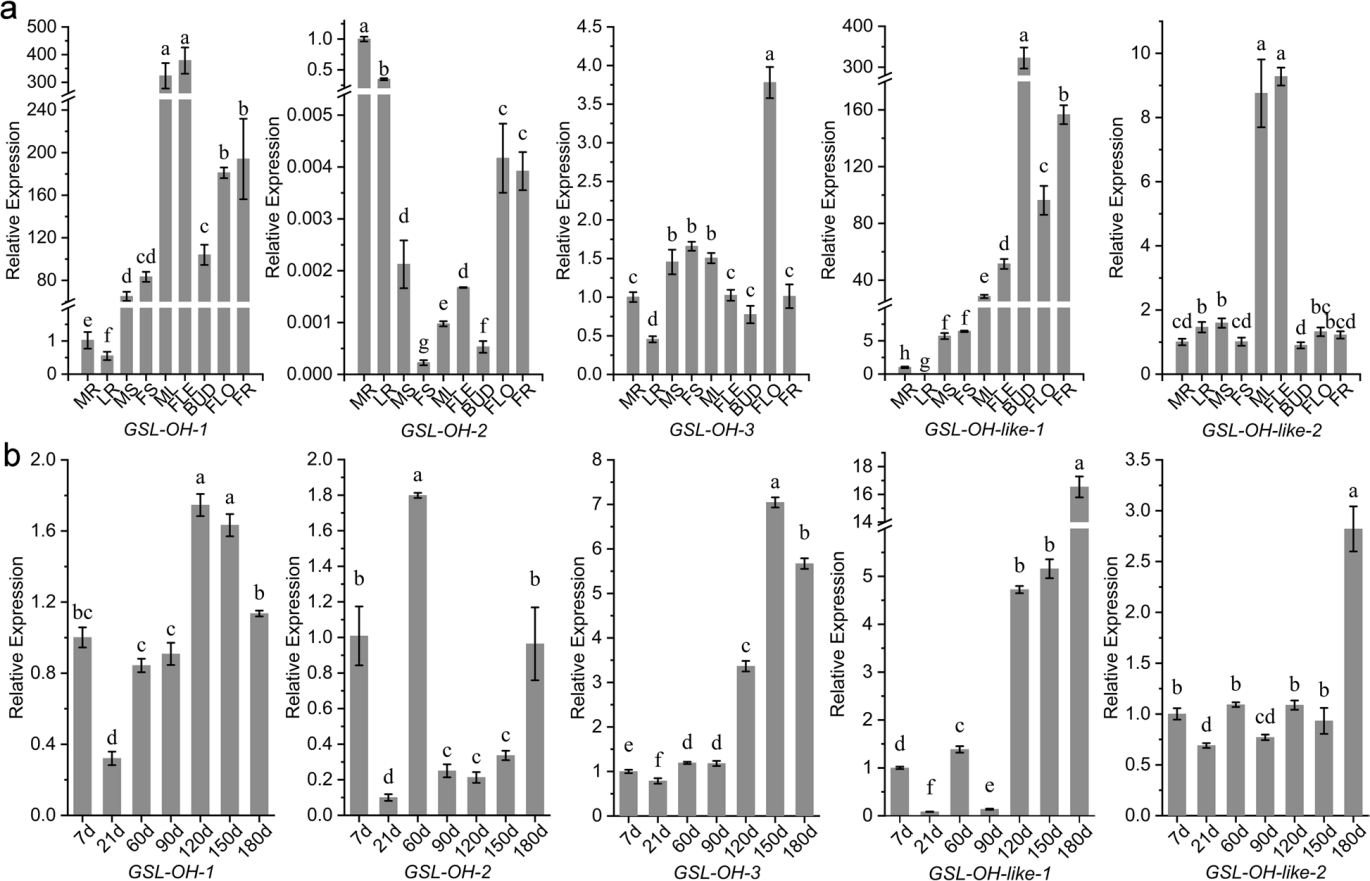
Gene expression patterns in different organs and developmental periods. **a** Different organs (MR: main roots, LR: lateral roots, MS: mature stems, FS: fresh stems, ML: mature leaves, FLE: fresh leaves, BUD: buds, FLO: flowers, FR: immature fruits); **b** Different developmental periods (7, 21, 60, 90, 120, 150 and 180 DAG); The determination results of the main roots and 7 DAG seedlings were chosen as the reference points, respectively. Each lowercase letter represents a distinctively significant level

To understand the expression characteristics of GSL-related genes, the expression patterns of the aliphatic GSL side chain modification genes were analyzed for nine different organs and seven developmental periods (Fig. 6), using *eIF2* and *PP2A-4* as the reference genes [61]. GSL-OH genes could catalyze alkenyl GSLs (i.e., GNA) to hydroxyalkenyl GSLs (i.e., PRO and EPI) and were homologous to *AT2G25450*. Three GSL-OH genes and two GSL-OH-like genes in tandem arrangement were found in *I. indigotica*. As shown in Fig. 6, the expression levels of *GSL-OH-1* were unstable in different developmental periods, varying from 0.32- to 1.75-fold, compared to the samples at 7 DAG, while the levels of *GSL-OH-2* were relatively low at 21 DAG (0.10-fold), 90 DAG (0.24-fold) and 150 DAG (0.21-fold). *GSL-OH-3* (over 3.39-fold) and *GSL-OH-like 1* (over 4.76-fold) exhibited higher expression levels after 120 DAG, while *GSL-OH-like 2* had the highest expression level (2.81-fold) at 180 DAG. Significant expression differences were also found in different organs. *GSL-OH-1* was mainly expressed in aboveground organs, while *GSL-OH-2* was expressed in roots. *GSL-OH-3* was highly expressed in flowers (3.78-fold) but showed lower levels in other organs (from 0.45-fold in lateral roots to 1.66-fold in fresh stems compared with main roots). In addition, *GSL-OH-like 1* and *GSL-OH-like 2* were mainly expressed in reproductive organs (over 96.33-fold) and leaves (over 8.75-fold). In short, the expression levels of five genes homologous to *GSL-OH* showed differences in different organs and developmental periods, which could lead to the subtle regulation of hydroxyalkenyl GSL biosynthesis in *I. indigotica.*

Generally, the ratio of the nonsynonymous substitution rate (Ka) to the synonymous substitution rate (Ks) reflects the selection pressure of paired genes, and the Ks value can be used to estimate divergence time [62]. We calculated Ka and Ks between *GSL-OH-1*/*GSL-OH-2* and *GSL-OH-2*/*GSL-OH-3* by KaKsCalculator 2.0 [63]. Furthermore, the divergence time of these two gene pairs was estimated using the formula T = Ks/2λ, where T means divergence time and λ (mutation rate) was set as 1.5 × 10^− 8^ substitutions/site/year [64]. The results are listed in Table 5. *GSL-OH-1* and *GSL-OH-3* derived from *GSL-OH-2* approximately 3.5–2.8 million years ago (Pliocene). The Ka/Ks ratios of *GSL-OH-1*/*GSL-OH-2* and *GSL-OH-2*/*GSL-OH-3* were above 0.5 (Table 5), displaying weak positive selection during evolution [65].

**Table 5:** Nonsynonymous substitution rate and synonymous substitution rate between specific GSL-OH genes

Gene pairs	Nonsynonymous substitution rate (Ka)	Synonymous substitution rate (Ks)	Ka/Ks	Divergent time (Million Years)
*GSL-OH-1*/*GSL-OH-2*	0.05525	0.10331	0.53480	3.44
*GSL-OH-2*/*GSL-OH-3*	0.05390	0.08470	0.63636	2.82

## Discussion

### The diversity in Glucosinolate accumulation in *I. indigotica*

GSLs exhibit strong anti-insect, antipathogen and immunoregulatory effects in plants [7], and their side-chain structures affect their biological functions. For instance, 1-methyethyl GSL and 1-methylpropyl GSL improved the resistance of *Arabidopsis* to *Erwinia carotovorum* [66], while 4MOI3M activated the innate immune system [67, 68]. Thus, there could exist some mechanisms for GSL diversification [67, 68].

In this study, the dominant GSLs in *I. indigotica* were EPI and I3M, followed by PRO and GNA, which was different from those in other *Isatis* spp. [24], in which I3M and GNA showed higher contents. A recent study of GSL profiles in dried roots of *I. indigotica* identified 16 GSLs, including 12 aliphatic GSLs, 2 aromatic GSLs and 2 indole GSLs [50]. In our study, there were 10 identified GSLs, while six potential new GSLs still needed to be further investigated (Fig. S4). The different experimental materials (dried roots vs. seedlings), the different dosages (6 kg vs. 0.2 g) and the different plant lines could contribute to the different research results. Both of these results suggest that more aliphatic GSLs and fewer aromatic and indole GSLs were present in dried roots, but more indole GSLs were found in seedlings and may enrich the GSL profiles in *I. indigotica*.

Based on our results, the aliphatic GSL contents initially decreased and then increased during the development process in *I. indigotica,* similar to what occurs in *Arabidopsis* [69], *Brassica oleracea* var. *italica* [70] and *Armoracia rusticana* [71]. GSLs contain considerable amounts of sulfur and are mainly involved in primary metabolic processes, with some breakdown products functioning as allelochemicals [14], which might be the reason for the reduction in aliphatic GSL contents. Indole GSLs accumulated dramatically during the germination period, with contents (2.45 μmol/g at 7 DAG) that increased by 22 times as high as that in seeds (0.10 μmol/g), which was different from the results for *Isatis tinctoria* L., in which GSLs accumulated slowly during the first month [49]. Moreover, the dominant GSL was sulfoglucobrassicin in *I. tinctoria*, which was different from that in *I. indigotica*. In addition, the distribution patterns of GSLs in *I. indigotica* were similar to those in *Arabidopsis* [38], except that fresh leaves accumulated more GSLs than fresh stems, which supports the current theory on the optimal distribution of defense substances [72]. It was noteworthy that (R, S)-GIT was only detected before 28 DAG (Table S2b) and was lacking in immature fruits, indicating its roles in the early development of seedlings of *I. indigotica*, as it might accumulate in later stages.

For MeJA treatment, the contents of indole GSLs increased 12-fold (3.49 μmol/g at 6 h), and aliphatic GSLs only increased 5.4-fold (0.36 μmol/g), similar to *Brassica rapa* ssp. *chinensis*, in which 8-fold and 3-fold increases in aliphatic and indole GSLs were found [73]. Nevertheless, the contents of aliphatic GSLs were unchanged under MeJA treatment in *Arabidopsis*, *B. oleracea* var. *italica* [74] and *Eruca sativa* [39]. In addition, NaCl significantly induced the accumulation of aliphatic and indole GSLs in *I. indigotica,* and the peak values appeared after 3–6 h and 48 h, respectively. Moreover, the continuous cold treatment exhibited a significant effect on aliphatic GSL accumulation. The transcriptome data indicated that the genes involved in glucosinolate and tryptophan metabolic pathways could take part in the vernalization process in *Pak choi* [75]. When considering both the different developmental periods and organs, there could also be some GSL profile changes when the *I. indigotica* seedlings overwintered.

### Genes involved in Glucosinolate metabolic pathways in *I. indigotica*

A total of 132 genes involved in GSL metabolic processes were identified in *I. indigotica*. It seemed that *MAM3*, *AOP3* and *CYP79F2* were missing in *I. indigotica* compared to *Arabidopsis*. Similar elements were missing in *Aethionema*, *Brassica* and *Raphanus*, indicating that these genes were genus-specific (Table S6, Fig. S5, S6 and S7). Gene duplication and subsequent subfunctionalization are important for creating and expanding biochemical diversity in plants [58]. Here, 68 genes were single-copy, including the core enzymes CYP79B, CYP79F and CYP83. Moreover, *MAM1* and *GSL-OH* had three functional copies, which could adjust metabolite flow. A recent report showed that CYP79C gene family members could catalyze six different amino acids to their corresponding oximes in transgenic tobacco [20]. Nine out of 26 Brassicaceae species had one or two *CYP79C1* copies, while 66 genes homologous to *AtCYP79C2* were found in these 26 species, suggesting expansion during evolution (Table S5b, Fig. S6). It was shown that there were two CYP79C gene family members in the ancestor of Brassicaceae according to the phylogenetic trees, but why most species lost *CYP79C1* is worth discussing.

Thirteen TGG1/2 and three TGG4/5 homologous loci were found, with some pseudogene fragments neglected in *I. indigotica* (Fig. 3b, Table S3). These genes could code proteins with complete domains, including TFNEP and ITENG (Fig. S10). NSP and MBP, acting as cofactors in the GSL breakdown process, had many similar fragments. Chromosome replications can occur after gene duplication due to their linear arrangement, and several genes could be nonfunctionalized to avoid biochemical disturbance in plants [76]. The number of *TGG* genes was examined in other Brassicaceae species (Table S4). Sixteen and fifteen *TGG1/2* genes were discovered in *B. oleracea* and *B. nigra*, respectively, while 18 *TGG4/5* genes were discovered in *Camelina sativa*. However, when taking domain completeness into consideration, it was found that only 6, 4, and 11 of the genes maintained their complete functional domains. Nevertheless, these species went through whole genome duplication/triplication separately, and thus, it was clear that *I. indigotica* could have more GSL breakdown-related genes, even though their actual biological functions are unknown. It is worth noting that more functional fragment replications could exist in *I. indigotica*, especially considering the size of the genome. In addition, we used all-vs-all BLAST to identify the orthologs of 13 TGG2 and 3 TGG4 genes (Table S6), and *TGG2–13* and *TGG4–10* were thought to be the most likely ancestor genes. Interestingly, neither of them were in the dense segments in which most GSL breakdown genes were located (Fig. 3), and further analysis showed that these dense segments had no synteny among *I. indigotica*, *Megadenia pygmaea* and *Arabidopsis* (data not shown), suggesting an insertion event during genome evolution. Recent genome sequencing of *Scutellaria baicalensis* demonstrated that 6 loci on pseudochromosome 9 could encode the *CHS2* gene, which is involved in root-specific flavone biosynthesis [77]. Similar results were also found in *Senna tora*, where 15 *CHS-L* genes were tandemly arranged [78]. Moreover, studies on the β-glucosidase (BGLU) [79] and BURP domain gene families [80] also suggested multiplied tandem duplication events in *Morinda officinalis* and *Bruguiera gymnorrhiza*, respectively. The balance between gene birth and gene death is key to duplication events. Gene family expansion enhanced gene expression levels and influenced the balance of metabolic flux. The subsequent regulation of expression could lead to three different fates for repeated genes, namely, neofunctionalization, subfunctionalization or nonfunctionalization [76], which is beneficial to environmental adaptation, such as glyphosate resistance in *Kochia scoparia* [81]*.* As more genome data are released, the significance behind this phenomenon will be revealed.

Brassicaceae species share the same side-chain elongation (SE) and core structure formation (CF) processes in the GSL biosynthesis pathway [8]. In contrast, side-chain modification (SM) has expanded the GSL profiles of different species, leading to at least 89 GSLs being dispersed over the Brassicales [9]. Thus, relaxed selection is beneficial to the catalysis reaction on different GSL structures (Fig. 5). Similarly, aliphatic GSLs show significant differences in their side chains, such as the length of the side chain and saturation degree of carbon molecules; however, indole GSLs experience hydroxylation and methoxylation at fixed positions in most species [8], requiring a stronger selective pressure during evolution (Fig. S9b, with a median less than 0.12). Another interesting finding was the weaker selective pressure on typical myrosinases than atypical myrosinases (Fig. S9c). We identified the copy numbers of different myrosinases among 26 Brassicaceae species, and the numbers of *PEN2* and *BGLU28* homologs were lower than those of *TGG1/2/3* and *TGG4/5/6*, two kinds of typical myrosinases. In particular, *PEN2,* a gene involved in the innate immune response to pathogens [68], remained single-copy in 20 out of 26 species (also one functional copy in *I. indigotica*), even in *B. oleracea* and *B. nigra*, two species that underwent a recent whole triplication event. Thus, the discrepancy in the Ka/Ks ratio between typical and atypical enzymes might reflect relaxed selection on duplicated genes, potentially leading to neofunctionalization during evolution [82, 83].

### Relationships between gene expression and Glucosinolate accumulation in *I. indigotica*

A pair of chiral isomers, goitrin and epigoitrin, showed differences in their activities [4, 13, 84]. Goitrin and epigoitrin are derived from progoitrin and epiprogoitrin, respectively [85]. Goitrin results in a goitrogenic reaction, but epigoitrin does not [86]. The GSL-OH homologous genes *GSL-OH-1*, *GSL-OH-2* and *GSL-OH-3* were found in *I. indigotica*. We tried to determine whether the different genes could catalyze one of the isomers, similar to RHO and SHO in *Barbarea vulgaris* [25]. Association analysis (Table S8) revealed that there were no apparent correlations between the gene expression levels and GSL accumulation, suggesting that the corresponding proteins encoded by those genes could catalyze PRO and EPI synthesis. Nevertheless, the transport of GSLs and breakdown of epiprogoitrin could not be fully excluded. To some extent, subfunctionalization could be considered since GSL-OH homologous genes showed organ-specific expression patterns (Fig. 6).

## Methods

### Plant materials and treatments

Seeds of *I. indigotica* purchased from Shaanxi Geo-Authentic Medicinal Plant Co. Ltd. (Xi’an, China) were cultivated in round pots (three seedlings per pot) in the greenhouse (25 ± 2 °C, 16 h light/8 h dark) until reaching different developmental stages and under different elicitor treatments; plants were watered every 2–3 days and maintained at 60–80% relative humidity of the soil. Different organs, namely, main roots, lateral roots, mature stems, middle stems, fresh stems, mature leaves, fresh leaves, buds, flowers and immature fruits, when flowers and fruits appeared simultaneously (in April, 2018), were samples from plants growing in the experimental field to provide the different organ samples (Table S9). For GSL content determination at different developmental stages, whole plants at 7, 14, 21, 28, 60, 90, 120, 150 and 180 days old were collected. The elicitor treatments, including MeJA (500 μmol/L), silver nitrate solution (AgNO_3_, 10 mmol/L), yeast extract (YE, 10 g/L), SA (300 μmol/L), sodium chloride solution (NaCl, 0.1 mol/L) and abscisic acid (ABA, 1 mmol/L), were conducted by foliage spraying. In addition, low temperature (4 °C) and mechanical damage (punching holes on leaves) were also used. All the plant materials were collected and put in liquid nitrogen immediately and then stored at − 80 °C for further analyses.

### Glucosinolate extraction and HPLC analysis

The extraction method was used with few modifications [87]. The plant materials were ground thoroughly in liquid nitrogen and then briefly put into a microtube with 5.0 mL precooled methanol/water (85:15, v/v) for deactivating myrosinase. After vortexing and standing for 30 min, the microtube was placed on a shaker for another 30 min. Thereafter, the extract solution was centrifuged at 4 °C and 8000 rpm for 5 min. And 40 μL of internal standard solution (sinigrin, 1.0 mg/mL, Sigma Sci. Co. Ltd.) was added to the extract solution and then stored at − 20 °C. Subsequently, the stored solution was slowly added to the DEAE Sephadex A-25 anion-exchange column (1.0 mL, Solarbo, Beijing) and then washed with 2.0 mL of sodium acetate solution (0.02 mol/L) three times and 2.0 mL of ultrapure water twice. After that, 500 μL of sulfatase solution (2.2 U/mL) was added to fill the whole column, and the column was kept at 35 °C for 16 h for complete desulfurization. Finally, the desulfo-GSLs were washed with 500 μL of ultrapure water three times.

The analysis of GSLs was performed on LC-2030 high-performance liquid chromatography (HPLC) equipment (Shimadzu, Japan) with an Inersil ODS-3 column (150 mm × 3.0 mm i.d., 3.0 μm, GL Sciences, Japan). The program was set as follows: from 0 to 17 min, 98% ultrapure water and 2% acetonitrile (Merck, Germany), which gradually changed to 80 and 20%, respectively, and held on for 3 min. Then, the percentage of ultrapure water was reduced to 70% in the next 5 min. Next, the column was washed with pure acetonitrile for 6 min and returned to 2% in the final step. The flow speed was set to 0.4 mL/min with a column temperature of 30 °C, each injection was 10 μL, and the UV detector wavelength was set to 229 nm. The peak areas were integrated to calculate the GSL contents by the internal standard method, and the correction factors were determined according to ISO 9167-1 [88]. The correction factors for other GSLs were 0.25 for indole and 1 for aliphatic GSLs according to Grosser and Van Dam (2017) [89].

The following formula was applied to calculate GSL contents:

$${w}_{measure}=\frac{k_{measure}}{m_{sample}}\times \frac{A_{measure}}{A_{IS}}\times \frac{c_{IS}\times {V}_{IS}}{M_{IS}}\times {10}^3$$

*w*_*measure*_, *k*_*measure*_ and *A*_*measure*_ indicate the content (μmol/g), relative correlation coefficient and HPLC peak area of a measured GSL. *A*_*IS*_, *c*_*IS*_, *V*_*IS*_ and *M*_*IS*_ represent the HPLC peak area of the internal standard (sinigrin), concentration of the internal solution (mg/mL), volume of the internal standard solution (mL, here 0.040 mL) and relative molecular mass of the internal standard (sinigrin, M = 397.5 g/mol), respectively. *m*_*sample*_ is the weight of raw materials used for extraction.

### Glucosinolate identification and determination

An Agilent 1200 HPLC system (Agilent, USA) with electrospray ionization coupled to an Agilent 6460 triple quadruple mass spectrometer (LC-ESI-MS/MS) was used to confirm the structures of the GSLs. The HPLC conditions were the same as those mentioned in the previous section, and the mass conditions are listed in Table 6. Compounds with *m*/*z* = 75 and featuring [M-G-H]^−^ molecular ion peaks were selected as candidate compounds [90, 91]. The positive ion peak, such as [M-G + H]^+^, was used for identification [51, 92]. For MS/MS conditions, the fragmentor voltage was optimized by approximately 1/3 of the molecular weight, and the collision energy number was set to approximately 1/15 of the given molecular weight.

**Table 6:** LC-MS/MS conditions

Items	Parameters
Gas Temp	300 °C
Gas Flow	10 L/min
Nebulizer	45 psi
Sheath Gas Temp	350 °C
Sheath Gas Flow	11 L/min
Capillary	3000 V(+)/3000 V(−)
Nozzle Voltage	500 V(+)/500 V(−)

### Identification of Glucosinolate metabolism-related genes

The genome of *I. indigotica* was independently sequenced on the Pac-Bio platform by our group, and the raw data were submitted to the National Center for Biotechnology Information (NCBI) database under BioProject PRJNA612129. The details of the assembly and annotation will be reported in another article. For short, the reads were filtered and then assembled with the help of Canu [93], WTDBG [94] and Falcon [95]. Optimization of the first-round assembly was performed by Quickmerge [96]. The Illumina sequencing result was merged to polish the assembly before using a high-throughput chromosome conformation capture technique (Hi-C) library, which was used to perform chromosome anchoring. Three strategies, namely, ab initio prediction, homologous prediction and transcriptome-guided prediction, were used for gene model fitting.

The GSL metabolism-related genes from *Arabidopsis* and *B. rapa* were obtained from TAIR [56] and BrassicaDB [57], respectively. A library was built, and the BLASTn program [97] was applied to identify the homologous genes in *I. indigotica* with a threshold of 1e-5. The sequences were corrected according to the transcriptome dataset to remove incorrect splicing predictions. Moreover, PFAM [98] and CDD [99] searches were conducted to ensure that the genes included conserved domains. The ExPASy tool [100] and Euk-mLoc2 [101] were used to predict the physical and chemical properties and protein sublocalization. MapChart 2.3.2 [102] was used to draw the distribution figure of GSL metabolism-related genes, while DNAMAN (Lynnon Corporation, Canada) was chosen to draw the figure of the sequence alignment results.

### Analysis of genes related to Glucosinolate metabolism

The *duplicate_gene_classifier* package in MCScanX [103] was used to determine the duplication type for glucosinolate metabolic genes. All-vs-all BLASTp [97] was performed for the *I. indigotica* genes using the parameters “blastp -evalue 1e-20 -outfmt 6 -num_alignments 6”, and the matchings with the genes themselves were removed. Then, the duplication type was determined using default parameters in the *duplicate_gene_classifier* package.

The identified glucosinolate metabolic genes from *I. indigotica* and the reported genes from *Arabidopsis* were used as queries to perform BLASTp searches in 24 Brassicaceae species protein databases (Table S6). Then, the best two hits from each species were BLASTp searched against all the proteins from *I. indigotica* and *Arabidopsis*. The bidirectional matching pairs were selected and regarded as possible homologous gene pairs from different species and were used as input for the ParaAT workflow [60] to obtain nonsynonymous nucleotide substitution rates (Ka) and synonymous nucleotide substitution rates (Ks). The result was filtered to remove gene pairs with a *p* value greater than 0.05. Violin plots were drawn with the help of the *ggplot2* package in R 4.1.1 [104].

Phylogenetic tree and orthologous gene analysis indicated that *GSL-OH-2* was the progenitor of *GSL-OH-1* and *GSL-OH-3* (Table S6, Fig. S5). Thus, Ka and Ks values were calculated between these two pairs using KaKsCalculator 2 software [63]. Divergence time was estimated according to the formula T = Ks/2λ, in which T was the divergence time (Mya) and λ was the substitution rate of nucleotides (rate/site/year). The λ value was set to 1.5 × 10^−8^, which is a frequently used mutation rate in Brassicaceae [64].

### Construction of the phylogenetic tree

The sequences of GSL metabolism-related genes were downloaded from the TAIR, BrassicaDB, NCBI [105], Ensemble [106] and JGI [107] websites in October 2021. All sequences were aligned by the Muscle program [108] with default parameters. The maximum likelihood method using the Jones-Taylor-Thornton (JTT) substitution model was applied for phylogenetic tree construction by FastTree 2.1.11 with the following parameters: “-pseudo -spr 4 -mlacc 3 -slownni -slow -gamma -no2nd” [109]. Phylogeny tests were verified by the bootstrap method with 1000 replications. All results were visualized by MEGA 7.0 [110].

### Gene expression analysis

To investigate the expression patterns of GSL metabolism-related genes, qRT-PCR was performed with TB Green® Premix Ex Taq™ Kit (Takara, Dalian, China) on a Roche LightCycler® 96 platform using a GSL side-chain modification gene (*GSL-OH*) as the example. The plant material was the same batch that was previously used in GSL determination;, samples were ground into powder in liquid nitrogen and then RNA was extracted according to the manual of the HiPure Plant RNA Mini Kit (Magen Technology, Guangzhou, China). The first chain of cDNA was generated by PrimeScript™ RT Master Mix (Takara, Dalian, China). The primers are shown in Table 7, with *IiPP2A-4* and *IieIF2* selected as the reference genes for the different periods and 5 tissues, respectively [61]. A three-step procedure was designed for qRT-PCR detection as follows: premelting at 95 °C for 30 s, 45 cycles of melting at 95 °C for 10 s, annealing at 55 °C for 10 s, and chain extension at 72 °C for 20 s, followed by signal acquisition of melting curves at 65 °C for 60 s and 97 °C for 1 s. The 2^-ΔΔCt^ method was used to calculate relative gene expression, with the expression level of the main root (for organs) or 7 DAG (for periods) set as 1 for reference. Each reaction was performed in three individual wells (*n* = 3), and ANOVA was used for statistical analysis (details in “Statistical Methods”).

**Table 7:** Primer sequences of the selected GSL side-chain modification genes in *I. indigotica*

Primer Name	Forward Primer (5′-3′)	Reverse Primer (5′-3′)	Length of Products (bp)	Related Gene Name
eIF2	TACCAGTGGCTCGCTTGAC	CAACCAAAGCAAATGACGTACTC	117	*eIF2*
PP2A-4	GAATGCCTGCGAAAGTATGG	TCCTAATGTTGTCAAGGGTCTC	151	*PP2A-4*
18849–1	CATATTCCATAACCCGCAGG	GTTACTGTAATACTTCACCAGCTGT	312	*GSL-OH-1*
18849–2	CCTAAACCTTCCTCGGTGGT	CGTCTTTAAGCTCCGCAACA	106	*GSL-OH-2*
7910	GCTGGTGAAGTATAGCAGTAACTC	CGCAACCCTAAAGCTTCTGAT	207	*GSL-OH-3*
13981	ATTACTACCCGCCTTGTCCA	CGGTTCTTCGCCTCTGTTC	228	*GSL-OH-like-1*
Tri14631	GGGCTCTTCTCGTCAACCT	GCGTGTACTCAGTGACGGTAG	251	*GSL-OH-like-2*

### Statistical methods

Every experiment was performed in triplicate with three biological replicates (n = 3), including GSL content determination and qRT-PCR analysis of gene expression. The GSL contents and gene expression levels are presented as the means ± standard errors (SEs), and they were assessed with one-way analysis of variance (ANOVA), followed by a Bonferroni correction for multiple tests. The significance levels (*p* < 0.05) are distinguished by different lowercase letters. For Ka/Ks ratio analysis, the Kruskal-Wallis H test (nonparametric test) was conducted, and a Bonferroni correction was used to adjust the *p* value (originally set as 0.05 for significance levels). The results of gene expression and the content determinations of progoitrin (PRO) and epiprogoitrin (EPI) were analyzed by Pearson’s correlation analysis. All statistical methods were performed by SPSS 22.0.

## Conclusions

In this study, GSL profiles and accumulation patterns in *I. indigotica* were studied. Ten GSLs were identified, including 5 aliphatic GSLs, 4 indole GSLs and 1 aromatic GSL, with the dominant GSLs being EPI, I3M and PRO. The total GSL contents varied across different development periods, organs, and elicitor treatments, indicating variable GSL accumulation. The reproductive organs accumulated more GSLs, and MeJA induced a 10.7-fold change after 6 h of treatment. A total of 132 genes involved in GSL metabolic processes were explored, and the divergence of the metabolic genes could lead to GSL profile differences. Relaxed selection was observed in side-chain modification genes, cofactors involved in GSL breakdown and transcription factors belonging to GSL metabolic pathways. The expression pattern of tandemly duplicated genes, the most common type of GSL-related gene, suggested neofunctionalization and subfunctionalization during evolution when the *GSL-OH* gene family was considered, while pseudogenes indicated nonfunctionalization during gene evolution. In conclusion, our study is the first to show GSL variations under different conditions and the metabolic pathways in *I. indigotica*, laying a firm foundation for the study of the accumulation and regulation of GSLs.

## Supplementary Information

**Additional file 1: Figure S1.** HPLC Chromatogram (229 nm) results of typical samples in *I. indigotica*. (a) Seeds (b) 14 DAG (c) Roots (d) 48 h low temperature treatment (e) Buds (f) 3 h MeJA treatment. Numbers represent: 1. desulpho-progoitrin (PRO); 2. desulpho-epiprogoitrin (EPI); 3. desulpho-sinigrin (SIN, internal standard); 4. desulpho-gluconapin (GNA); 5. desulpho-4-hydroxy-3-indolylmethyl GSL (4OHI3M); 7. desulpho-glucotropaeolin (GTL); 8. desulpho-Indolyl-3-methyl (I3M); 9. desulpho-4-methoxy-3- indolylmethyl GSL (4MOI3M); 10. desulpho-R,S-glucoisatisin (GIT); 11. desulpho-1-methoxy-3-indolylmethyl GSL (1MOI3M).

**Additional file 2: Figure S2.** The molecular fragments of mass spectrum under negative mode. Y-axis represents ion intensity, while numbers on X-axis are the mass-to-charge ratio (*m/z*). GSL name and corresponding retention time are shown on the upper right corner of every figure and the unit of the latter one is minute (min).

**Additional file 3: Figure S3.** The results of neutral loss of mass spectrum under negative mode. Y-axis represents ion intensity, while numbers on X-axis are the mass-to-charge ratio (*m/z*). All neutral loss mass (198 Da or 162 Da) and the mass-to-charge ratio of each desulpho GSLs are shown on the upper right corner of every figure.

**Additional file 4: Figure S4.** The mass spectrum images of six uncharacterized GSLs. Y-axis represents ion intensity, while numbers on X-axis are the mass-to-charge ratio (*m/z*). GSL name is shown on the upper right/left corner of every figure. (a) The fragments of complete desulpho molecules under negative mode (b) The neutral loss results under negative mode (c) The fragments of desulphurizated glycoside aglycone under positive mode.

**Additional file 5: Figure S5.** Phylogenetic trees of *AOP* (belonging to subgroup 20 of *2OGD* gene family) and *GSL-OH* (belonging to subgroup 31 of *2OGD* gene family). Three *2OGD* genes from *Oryza sativa* (http://rice.plantbiology.msu.edu/) are chosen as outgroup sequences. Different branches are distinguished with colors. And green, red, pink, yellow, black and gray circles represent sequences of *Arabidopsis*, *I. indigotica*, *Megadenia pygmaea*, *Barbarea vulgaris*, *A. arabicum* and *O. sativa*, respectively. Some sequences are removed because of skeptical alignments.

**Additional file 6: Figure S6.** Phylog enetic trees of certain CYP gene family members. Sequences of CYP51G in *I. indigotica* are chosen as global outgroups, where tree roots are put. *CYP81D* and *CYP71AN* members from *Arabidopsis* and *I. indigotica* are set to be out groups of *CYP81* and *CYP83*, respectively. Coloured circles represent sequences from specific species: green for *Arabidopsis*, red for *I. indigotica*, pink for *M. pygmaea*, black for *A. arabicum* and gray for *Carica papaya* or *Moringa oleifera* (two relatives of Brassicaceae). Some sequences are removed because of skeptical alignments.

**Additional file 7: Figure S7.** Phylogenetic trees of certain *MAM*-*IPMS* gene family members. Two genes coding isopropylmalate synthase (*IPMS*) from *Oryza sativa* (http://rice.plantbiology.msu.edu/) are chosen as outgroup sequences. Different branches are distinguished with colours. And green, red, pink, black and gray circles represent sequences of *Arabidopsis*, *I. indigotica*, *M. pygmaea*, *A. arabicum* and *Carica papaya* or *O. sativa*, respectively. Some sequences are removed because of skeptical alignments.

**Additional file 8: Figure S8.** An overview of homologous gene pairs identified in this study. Green cells represent the existence of gene pairs contrary to grey cells, which mean failure in identify homolog pairs. The raw data can be checked in Table S6. (A) Overview of gene pairs in *I. indigotica* (B) Overview of gene pairs in *Arabidopsis.*

**Additional file 9: Figure S9.** Ka/Ks ratio comparison between different subgroups in GSL metabolic pathway. The subgroup division and other details are given in Table S7. Significance level is set as 0.05 under Mann-Whitney U test. *, ** and *** represent *p* < 0.05, 0.01 and 0.001, respectively. (a) Comparison within three subgroups in GSL core structure formation. (b) Comparison between genes involved in aliphatic and indole GSL side-chain modification. (c) Comparison between atypical and typical myrosinases.

**Additional file 10: Figure S10.** The sequence alignment of beta-thioglucoside glucohydrolase proteins in *Arabidopsis* and *I. indigotica.* The colours of key motif regions are inverted to emphasize them. It shows that two motifs (motif 1 for acid/base catalyst and motif 2 for nucleophile) are all complete in beta-thioglucoside glucohydrolase proteins of *I. indigotica*, suggesting their ability to work as glycosidase.

**Additional file 11: Table S1.** LC-MS/MS fragment results of desulpho-GSLs.

**Additional file 12: Table S2.** The GSL accumulations in *I. indigotica* for the different periods, organs and treatments. All the data are shown as average contents ± standard deviation (*n* = 3). Lowercase letters behind the content data are used to indicate the significant levels. N.D.: Not detected.

**Additional file 13: Table S3.** The identified genes and subcellular localization results. The lists are in an alphabetic arrangement.

**Additional file 14: Table S4.** Identified *TGG* genes in 26 different Brassicaceae species. (a) Number of myrosinase gene homologs in 26 Brassicaceae species. (b) Homologous sequence names of myrosinase genes in 26 Brassicaceae species.

**Additional file 15: Table S5.** Homologous sequences of *AOP*, *GSL-OH*, *CYP79*, *CYP81F*, *CYP83*, *MAM* and *IPMS* genes in 26 Brassicaceae species.

**Additional file 16: Table S6.** Homolog gene pairs identified. Green is the background colour of cells, including the homologous genes. The bidirectional best hits are used as homologous pairs here, noting some may not be true orthologs with each other. (a) Identified orthologs of glucosinolate-related genes of *I. indigotica* in 25 Brassicaceae species (b) Identified orthologs of glucosinolate-related genes of *Arabidopsis* in 25 Brassicaceae species.

**Additional file 17: Table S7.** Raw data of Ka/Ks ratio calculation results. The ratio was coloured to show the variation tendency. Source genes are from *I. indigotica*, and query genes from other Brassicaceae species. Eight processes were divided according to the gene functions in glucosinolate metabolic pathways. For subgroup division, CYP79 and CYP83 are thought to be key enzymes in GSL biosynthesis. GGP, SUR and UGT74B are shared with both aliphatic and indole GSL biosynthesis processes, named “common genes”. Different members of GST and ST5 are linked to different GSL formation, and were grouped together. Atypical myrosinases include BGLU28, BGLU30, PEN2 and PYK10, contrast to typical myrosinase TGGs. For side-chain modification, FMO, AOP and GSL-OH take their parts in aliphatic GSL modification, while CYP81F and IGMT modify indole ring in the nature.

**Additional file 18: Table S8.** Pearson association analysis between the gene expression levels and GSL contents. The significant correlation is marked with an asterisk behind the number.

**Additional file 19: Table S9.** Selection criteria for samples of ten organs.

**Additional file 20: Supplementary Data 1.** Protein sequences of *AOP*,and *GSL-OH*, used for phylogenetic tree construction in this study. This is the source file for Fig. S5. Detailed information can be found in Table S5b.

**Additional file 21: Supplementary Data 2.** Protein sequences of *CYP79*, *CYP81F* and *CYP83* used for phylogenetic tree building in this study. This is the source file for Fig. S6. Detailed information can be found in Table S5c.

**Additional file 22: Supplementary Data 3.** Protein sequences of *MAM* and *IPMS* used for phylogenetic tree building in this study. This is the source file for Fig. S7. Detailed information can be found in Table S5d.

## Data Availability

The datasets supporting the conclusions of this article are included within the article and its additional files. Raw sequencing data were deposited in the Sequencing Read Achive database in NCBI (Accession ID: PRJNA612129), and predict sequences in this study were submitted to GenBank in NCBI with available accession number listed in Table S3.

## Abbreviations

GSL(s): Glucosinolate(s)
PRO: Progoitrin
EPI: Epiprogoitrin
GNA: Gluconapin
I3M: Indole-3-methyl GSL
GIT: Glucoisatisin
FW: Fresh weight
DAG: Day(s) after germination
h: hour(s)
2OGD: 2-oxoglutarate-dependent dioxygenase
NSP: Nitrile-specifier proteins
BGLU: β-glucosidase
TGG: Thioglucoside glucohydrolase
MBP: Myrosinase-binding protein
