# Supplementary figures and images for: Insights into glucosinolate accumulation and metabolic pathways in Isatis indigotica Fort
Source: BMC Plant Biol. 2022 Feb 22;22:78. doi: 10.1186/s12870-022-03455-6 (PMC8862337; doi:10.1186/s12870-022-03455-6)

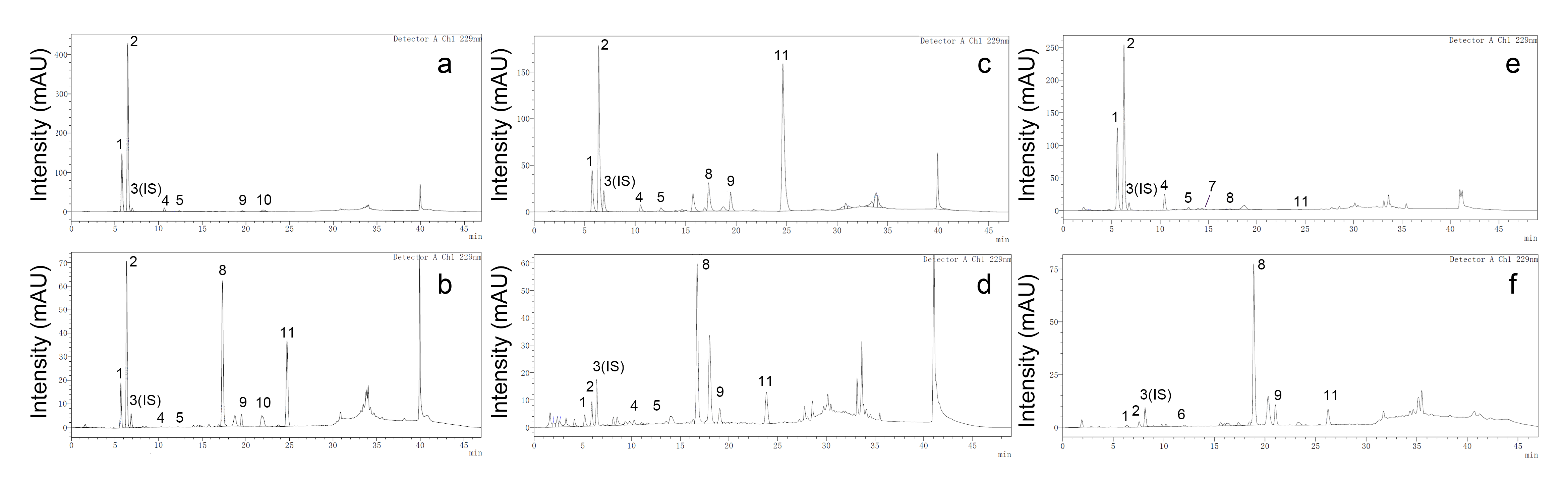

Supplement: Supplementary file 1 — Additional file 1: Figure S1. HPLC Chromatogram (229 nm) results of typical samples in I. indigotica. (a) Seeds (b) 14 DAG (c) Roots (d) 48 h low temperature treatment (e) Buds (f) 3 h MeJA treatment. Numbers represent: 1. desulpho-progoitrin (PRO); 2. desulpho-epiprogoitrin (EPI); 3. desulpho-sinigrin (SIN, internal standard); 4. desulpho-gluconapin (GNA); 5. desulpho-4-hydroxy-3-indolylmethyl GSL (4OHI3M); 7. desulpho-glucotropaeolin (GTL); 8. desulpho-Indolyl-3-methyl (I3M); 9. desulpho-4-methoxy-3- indolylmethyl GSL (4MOI3M); 10. desulpho-R,S-glucoisatisin (GIT); 11. desulpho-1-methoxy-3-indolylmethyl GSL (1MOI3M). [file 12870_2022_3455_MOESM1_ESM.tif]

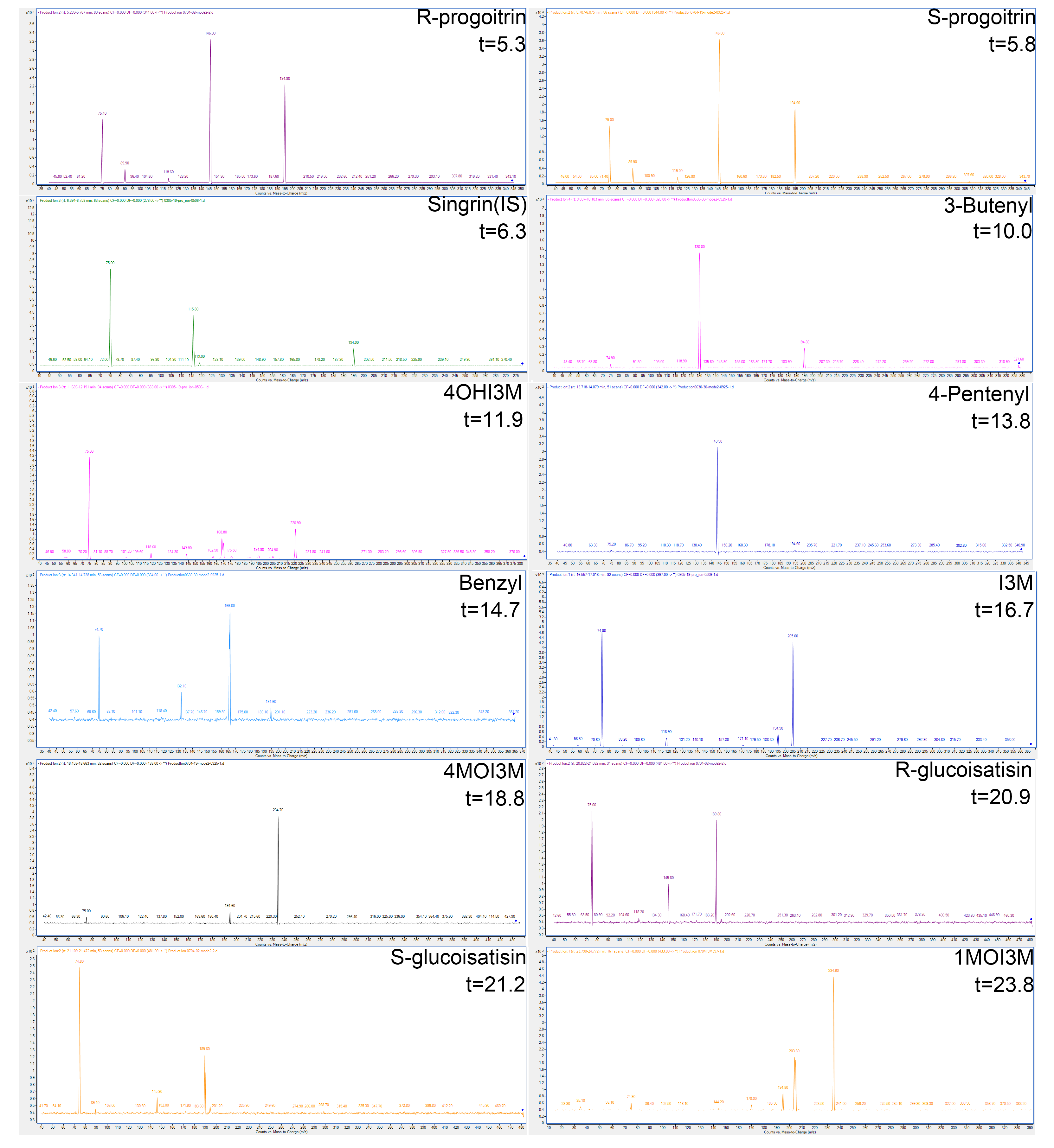

Supplement: Supplementary file 2 — Additional file 2: Figure S2. The molecular fragments of mass spectrum under negative mode. Y-axis represents ion intensity, while numbers on X-axis are the mass-to-charge ratio (m/z). GSL name and corresponding retention time are shown on the upper right corner of every figure and the unit of the latter one is minute (min). [file 12870_2022_3455_MOESM2_ESM.tif]

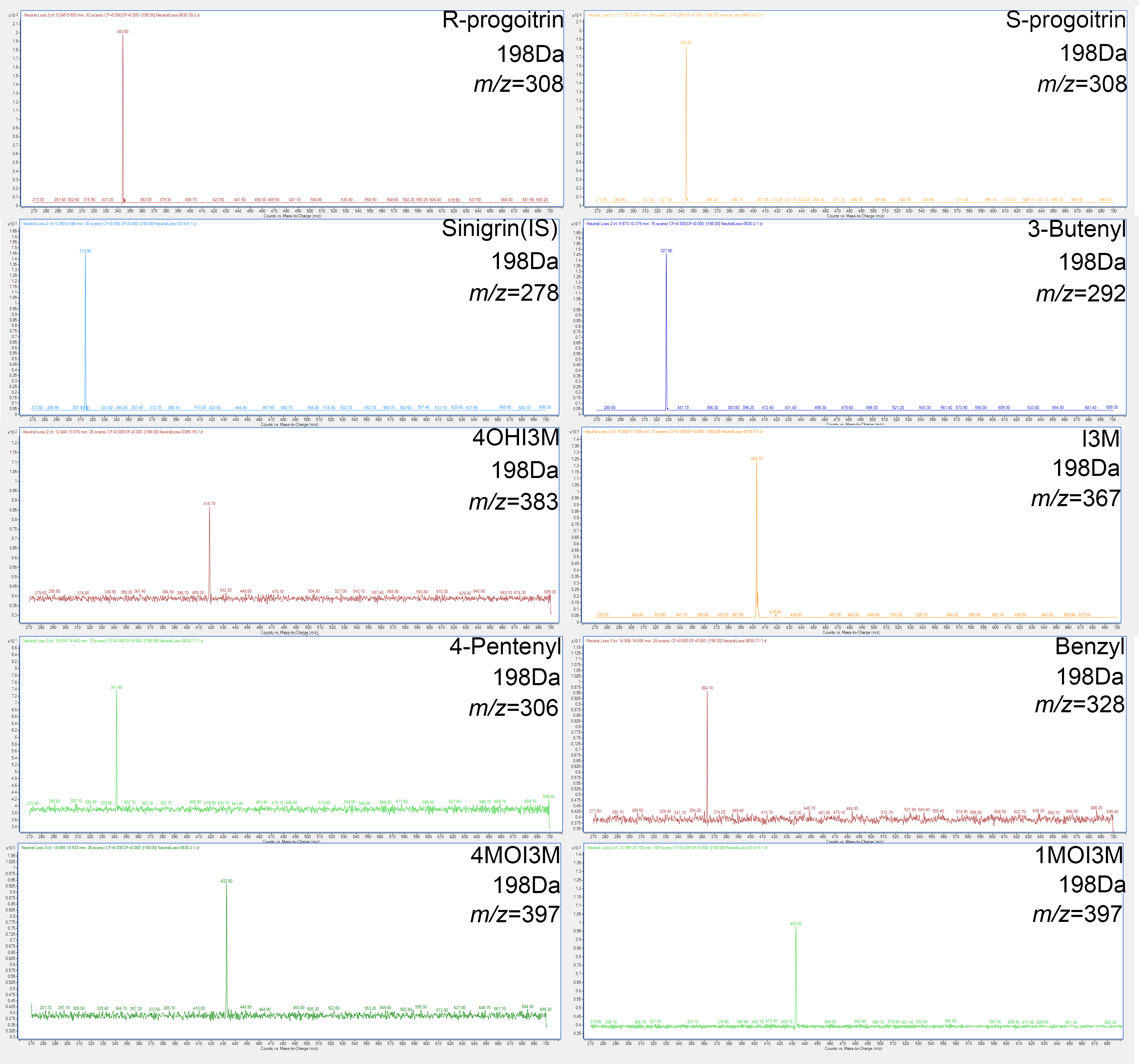

Supplement: Supplementary file 3 — Additional file 3: Figure S3. The results of neutral loss of mass spectrum under negative mode. Y-axis represents ion intensity, while numbers on X-axis are the mass-to-charge ratio (m/z). All neutral loss mass (198 Da or 162 Da) and the mass-to-charge ratio of each desulpho GSLs are shown on the upper right corner of every figure. [file 12870_2022_3455_MOESM3_ESM.tif]

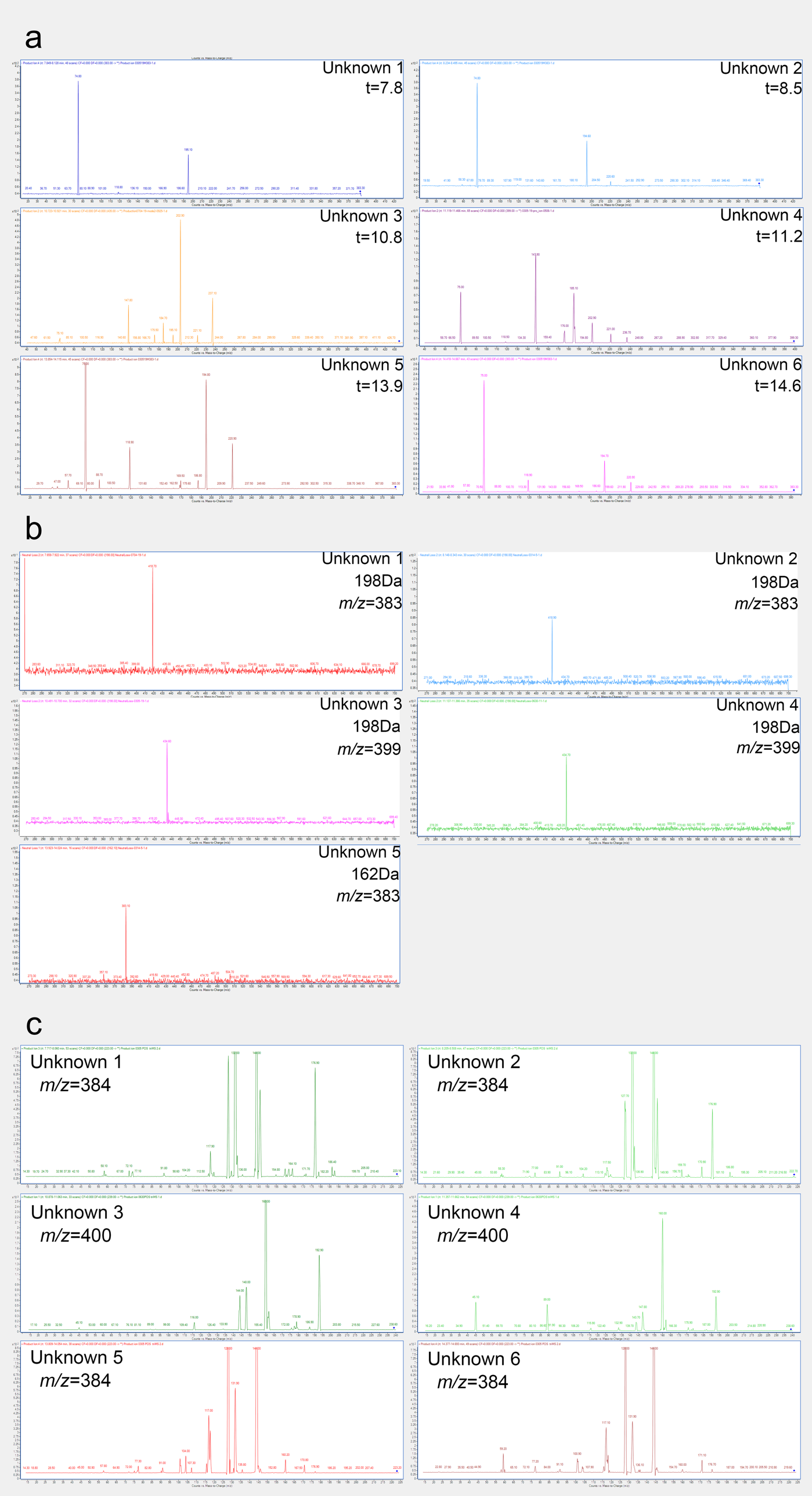

Supplement: Supplementary file 4 — Additional file 4: Figure S4. The mass spectrum images of six uncharacterized GSLs. Y-axis represents ion intensity, while numbers on X-axis are the mass-to-charge ratio (m/z). GSL name is shown on the upper right/left corner of every figure. (a) The fragments of complete desulpho molecules under negative mode (b) The neutral loss results under negative mode (c) The fragments of desulphurizated glycoside aglycone under positive mode. [file 12870_2022_3455_MOESM4_ESM.tif]

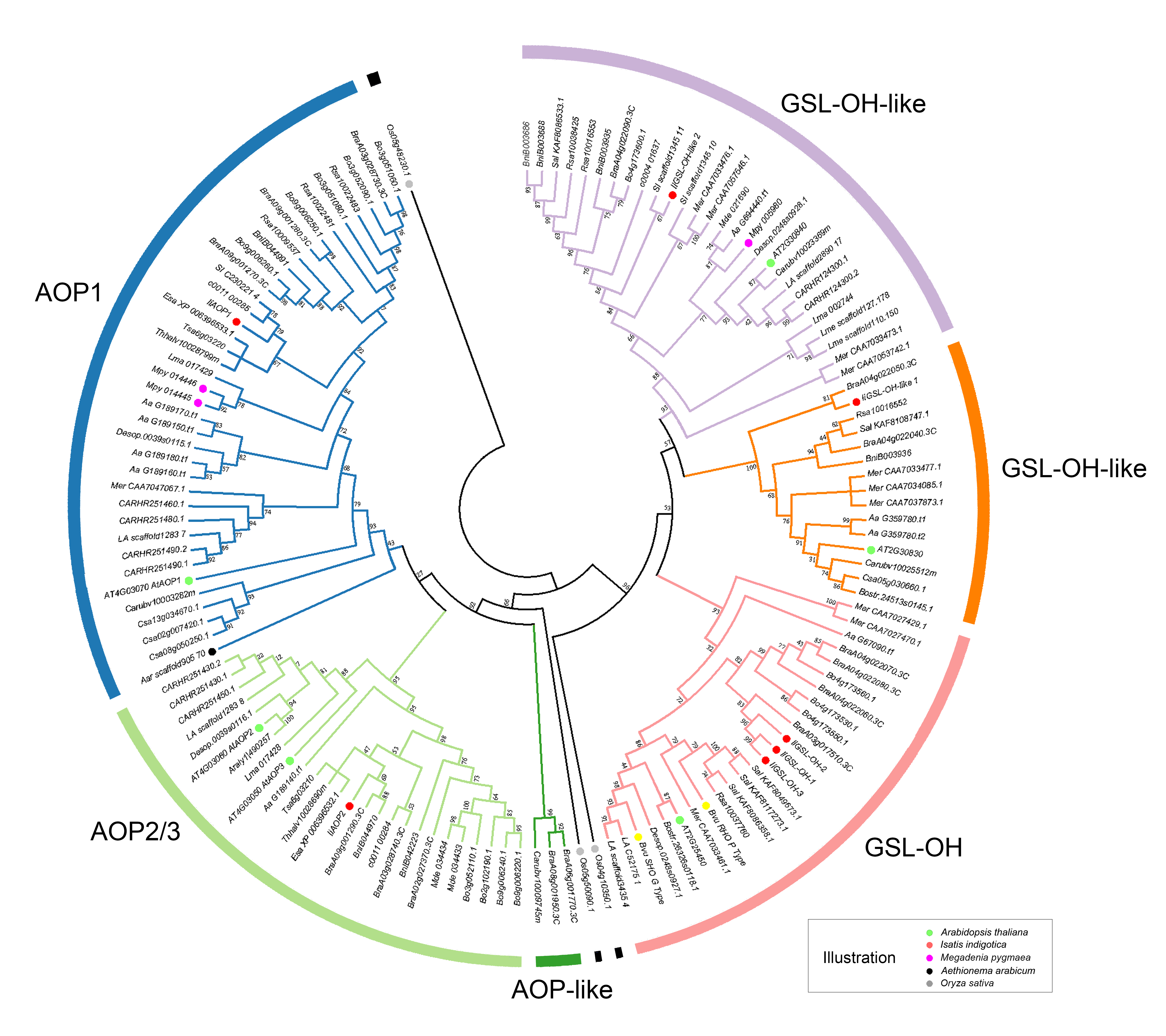

Supplement: Supplementary file 5 — Additional file 5: Figure S5. Phylogenetic trees of AOP (belonging to subgroup 20 of 2OGD gene family) and GSL-OH (belonging to subgroup 31 of 2OGD gene family). Three 2OGD genes from Oryza sativa (http://rice.plantbiology.msu.edu/) are chosen as outgroup sequences. Different branches are distinguished with colors. And green, red, pink, yellow, black and gray circles represent sequences of Arabidopsis, I. indigotica, Megadenia pygmaea, Barbarea vulgaris, A. arabicum and O. sativa, respectively. Some sequences are removed because of skeptical alignments. [file 12870_2022_3455_MOESM5_ESM.tif]

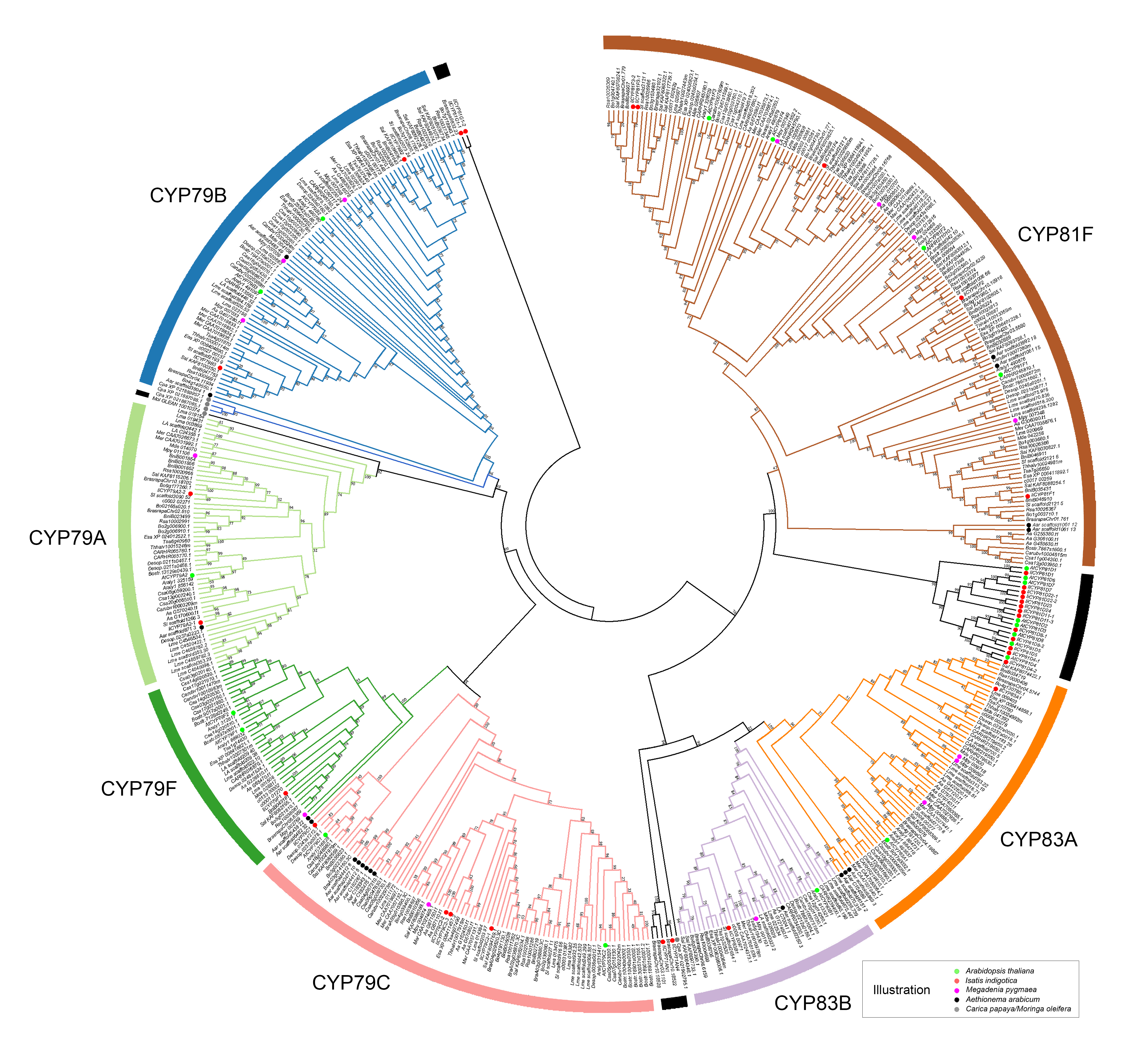

Supplement: Supplementary file 6 — Additional file 6: Figure S6. Phylog enetic trees of certain CYP gene family members. Sequences of CYP51G in I. indigotica are chosen as global outgroups, where tree roots are put. CYP81D and CYP71AN members from Arabidopsis and I. indigotica are set to be out groups of CYP81 and CYP83, respectively. Coloured circles represent sequences from specific species: green for Arabidopsis, red for I. indigotica, pink for M. pygmaea, black for A. arabicum and gray for Carica papaya or Moringa oleifera (two relatives of Brassicaceae). Some sequences are removed because of skeptical alignments. [file 12870_2022_3455_MOESM6_ESM.tif]

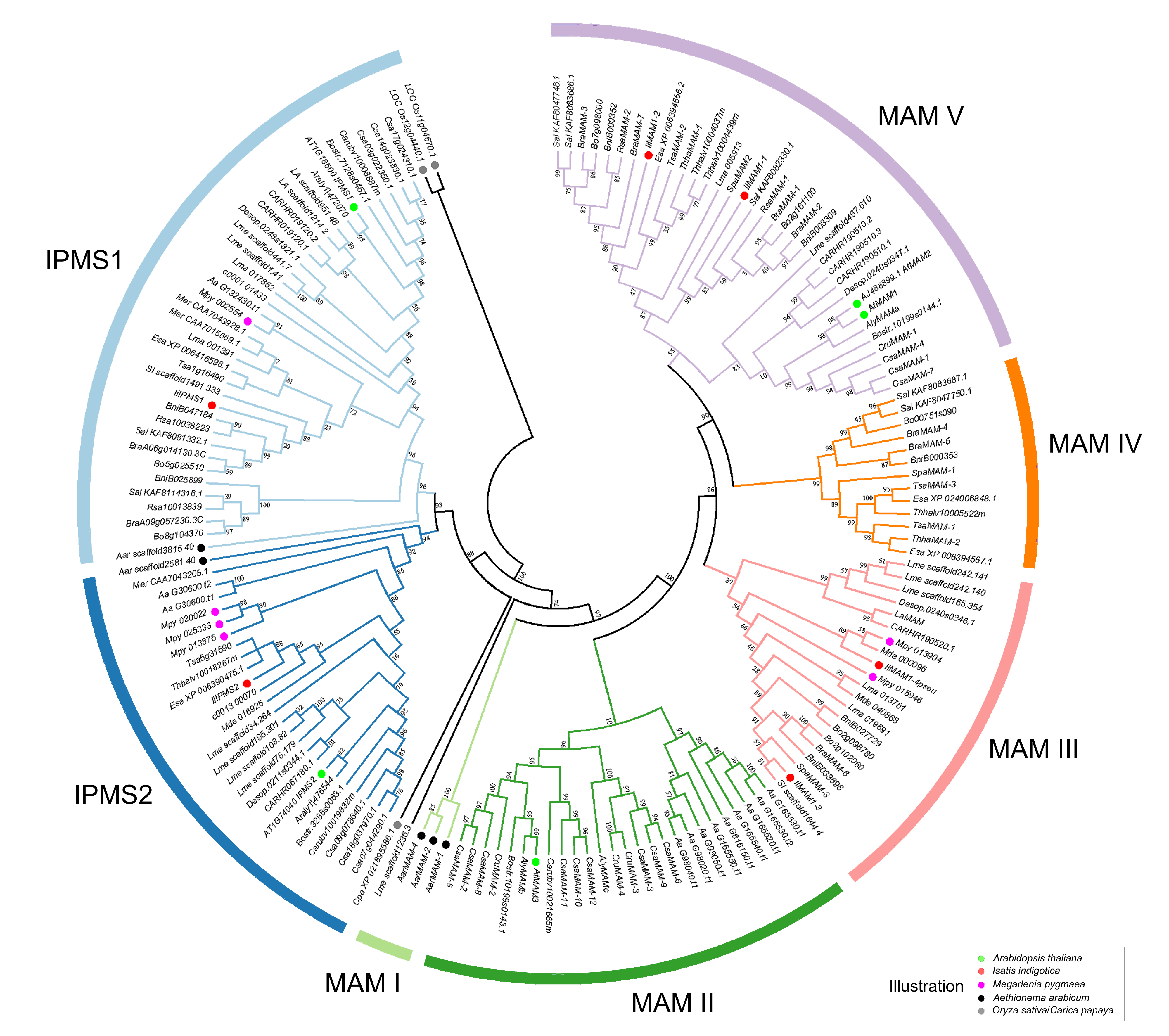

Supplement: Supplementary file 7 — Additional file 7: Figure S7. Phylogenetic trees of certain MAM-IPMS gene family members. Two genes coding isopropylmalate synthase (IPMS) from Oryza sativa (http://rice.plantbiology.msu.edu/) are chosen as outgroup sequences. Different branches are distinguished with colours. And green, red, pink, black and gray circles represent sequences of Arabidopsis, I. indigotica, M. pygmaea, A. arabicum and Carica papaya or O. sativa, respectively. Some sequences are removed because of skeptical alignments. [file 12870_2022_3455_MOESM7_ESM.tif]

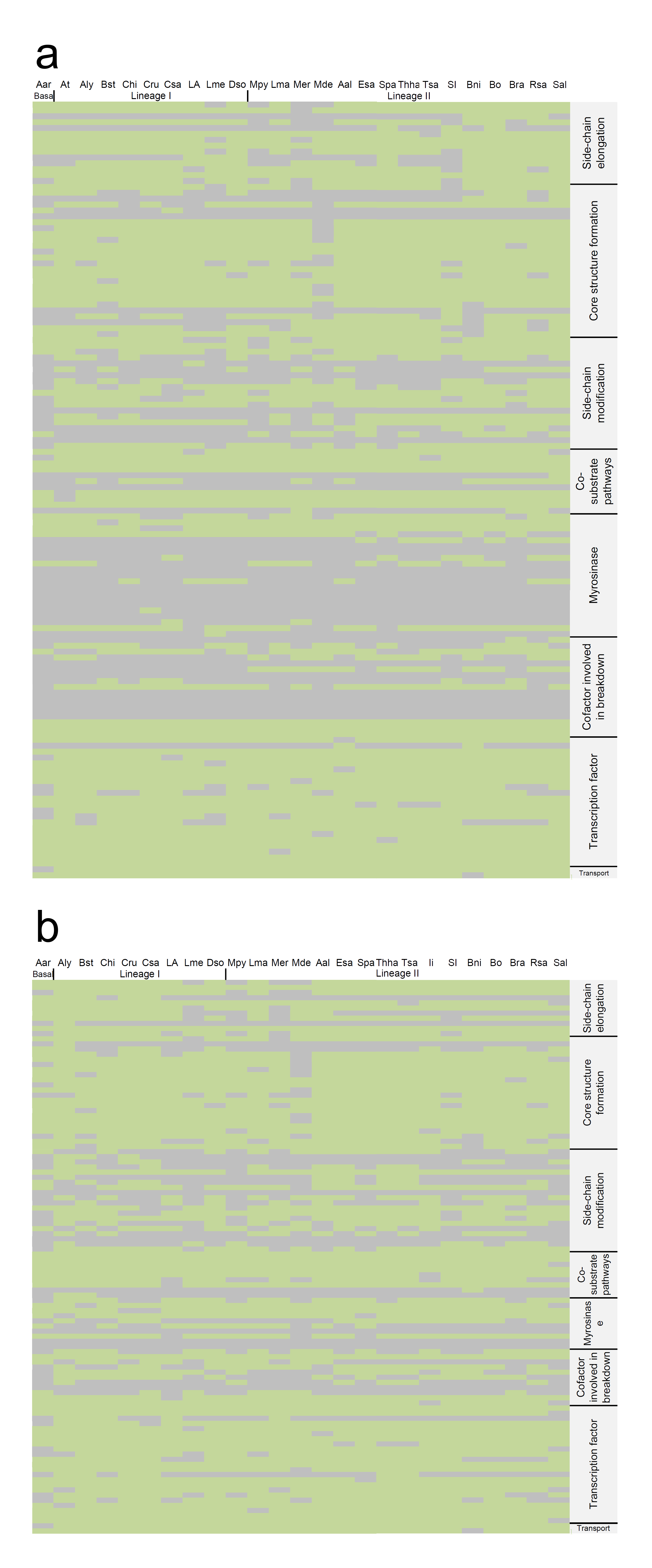

Supplement: Supplementary file 8 — Additional file 8: Figure S8. An overview of homologous gene pairs identified in this study. Green cells represent the existence of gene pairs contrary to grey cells, which mean failure in identify homolog pairs. The raw data can be checked in Table S6. (A) Overview of gene pairs in I. indigotica (B) Overview of gene pairs in Arabidopsis. [file 12870_2022_3455_MOESM8_ESM.tif]

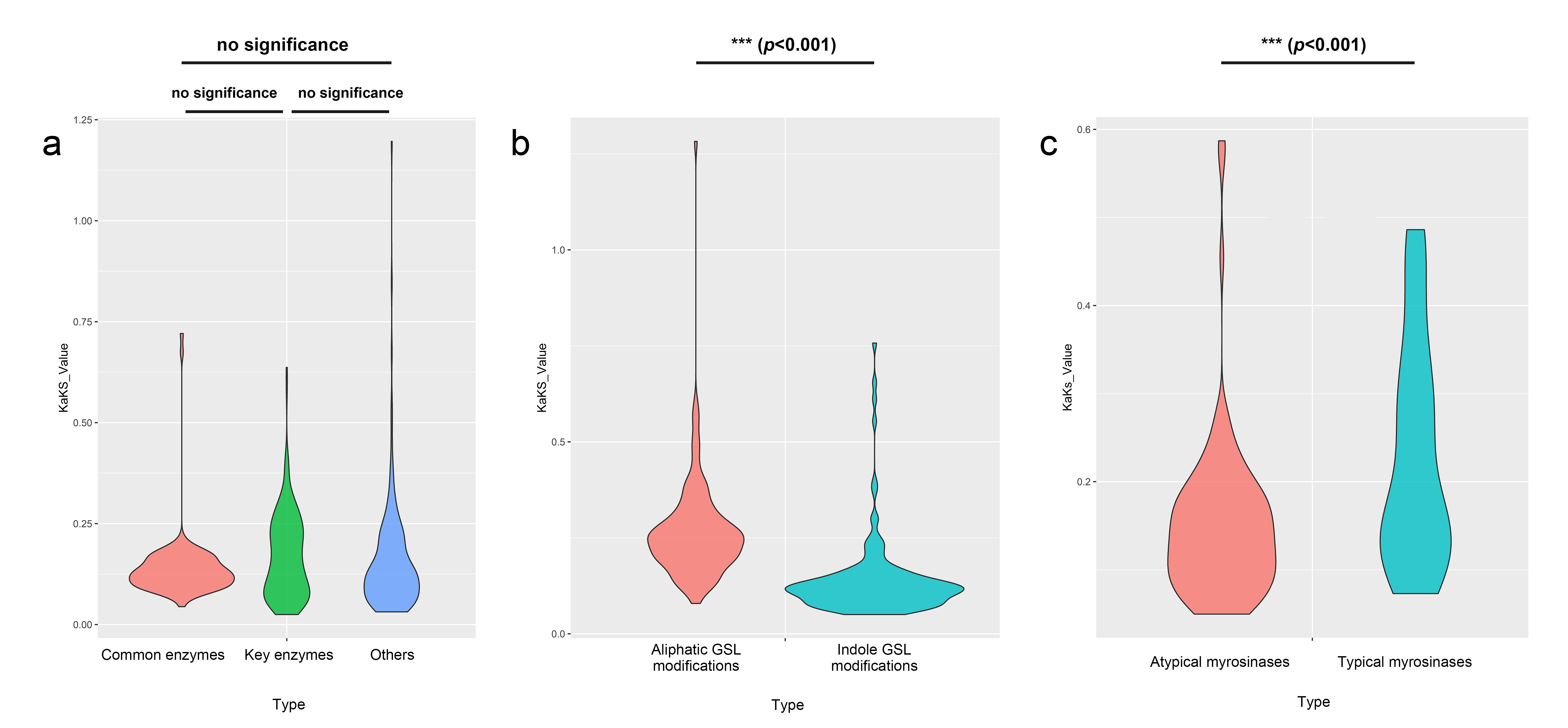

Supplement: Supplementary file 9 — Additional file 9: Figure S9. Ka/Ks ratio comparison between different subgroups in GSL metabolic pathway. The subgroup division and other details are given in Table S7. Significance level is set as 0.05 under Mann-Whitney U test. *, ** and *** represent p < 0.05, 0.01 and 0.001, respectively. (a) Comparison within three subgroups in GSL core structure formation. (b) Comparison between genes involved in aliphatic and indole GSL side-chain modification. (c) Comparison between atypical and typical myrosinases. [file 12870_2022_3455_MOESM9_ESM.tif]

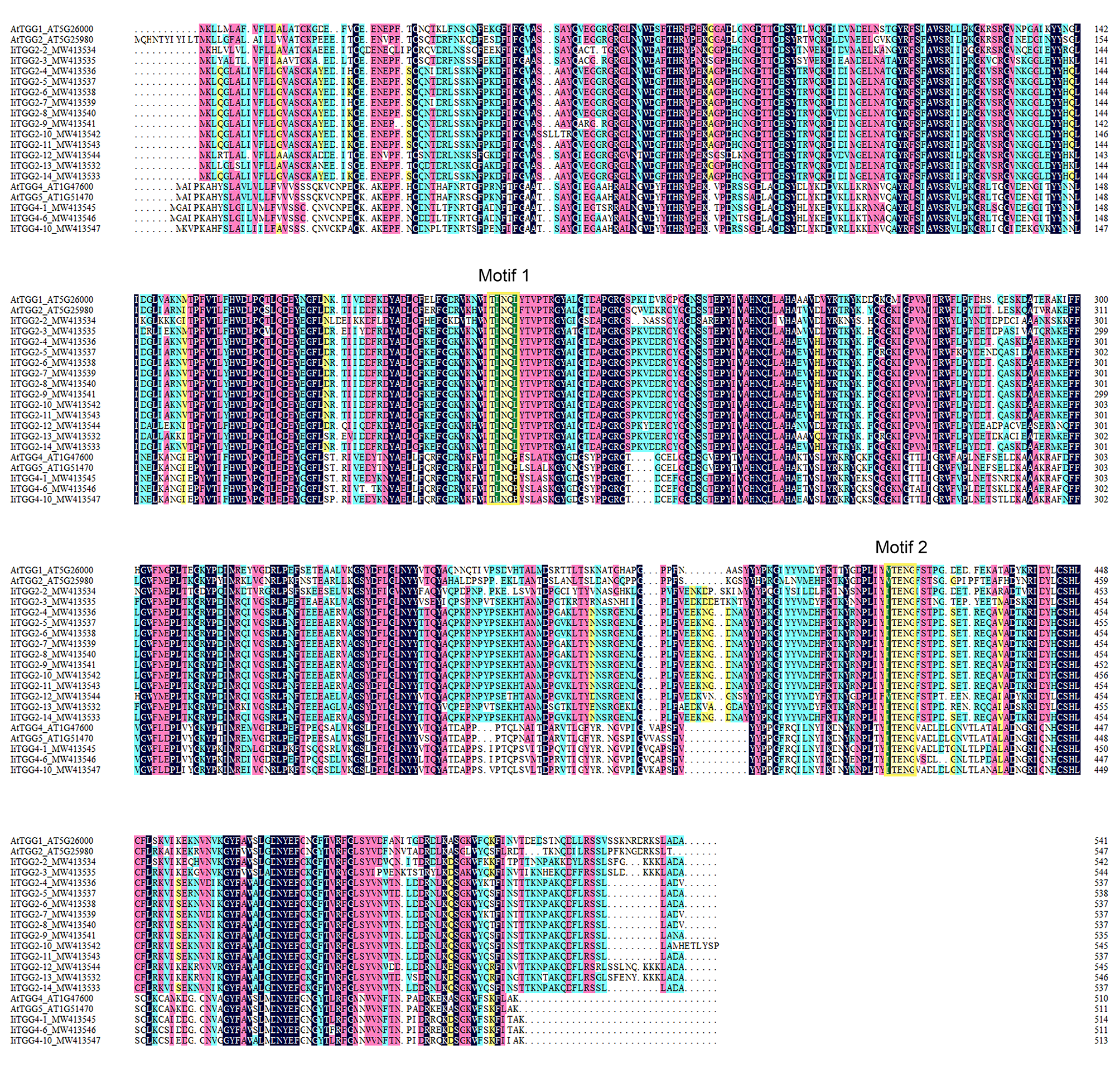

Supplement: Supplementary file 10 — Additional file 10: Figure S10. The sequence alignment of beta-thioglucoside glucohydrolase proteins in Arabidopsis and I. indigotica. The colours of key motif regions are inverted to emphasize them. It shows that two motifs (motif 1 for acid/base catalyst and motif 2 for nucleophile) are all complete in beta-thioglucoside glucohydrolase proteins of I. indigotica, suggesting their ability to work as glycosidase. [file 12870_2022_3455_MOESM10_ESM.tif]
