# Supplementary material for: Insights into glucosinolate accumulation and metabolic pathways in Isatis indigotica Fort
Source: BMC Plant Biol. 2022 Feb 22;22:78. doi: 10.1186/s12870-022-03455-6 (PMC8862337; doi:10.1186/s12870-022-03455-6)
Supplement: Supplementary file 12 — Additional file 12: Table S2. The GSL accumulations in I. indigotica for the different periods, organs and treatments. All the data are shown as average contents ± standard deviation (n = 3). Lowercase letters behind the content data are used to indicate the significant levels. N.D.: Not detected. [file 12870_2022_3455_MOESM12_ESM.docx]

**Table S2** The GSL accumulations in *I. indigotica* for the different periods, organs and treatments

All the data are shown as average contents ± standard deviation (n=3). Lowercase letters behind the content data are used to indicate the significant levels. The unit of all data is μmol/g (FW).

N.D.: Not detected.

**Table S2a**: Table of comparisons

| Chemical Name | Common Name | Abbreviation |
| --- | --- | --- |
| R-2-Hydroxy-3-butenyl GSL | Progoitrin | PRO |
| S-2-Hydroxy-3-butenyl GSL | Epiprogoitrin | EPI |
| 2-Propenyl GSL | Sinigrin | SIN |
| 3-Butenyl GSL | Gluconapin | GNA |
| 4-Hydroxy-3-indolylmethyl GSL | 4-Hydroxyglucobrassicin | 4OHI3M |
| 4-Pentenyl GSL | Glucobrassicanapin | GBN |
| Benzyl GSL | Glucotropaeolin | GTL |
| Indole-3-methyl GSL | Glucobrassicin | I3M |
| 4-Methoxy-3-indolylmethyl GSL | 4-Meyhoxyglucobrassicin | 4MOI3M |
| 3-Buten-2-yl-2-(2-oxoindolin-3-yl) acetate GSL | Glucoisatisin | GIT |
| 1-Methoxy-3-indolylmethyl GSL | Neoglucobrassicin | 1MOI3M |

**Table S2b** Glucosinolates accumulation data in different periods

**Table S2b.1**

|  | Seeds | 7d | 14d | 21d | 28d | 60d | 90d | 120d | 150d | 180d |
| --- | --- | --- | --- | --- | --- | --- | --- | --- | --- | --- |
| PRO | 6.75354±1.70981 ^c^ | 2.64762±0.89444 ^b^ | 1.08923±0.20094 ^ab^ | 0.59858±0.04097 ^ab^ | 0.44864±0.09408 ^ab^ | 0.09187±0.00647 ^a^ | 0.20027±0.04676 ^a^ | 0.15248±0.01338 ^a^ | 1.17598±0.16857 ^ab^ | 0.92576±0.16523 ^ab^ |
| EPI | 18.98566±4.76068 ^c^ | 8.69468±2.89803 ^b^ | 3.85028±0.73273 ^ab^ | 2.70189±0.19284 ^ab^ | 1.49503±0.1792 ^a^ | 0.38876±0.00305 ^a^ | 0.38608±0.05886 ^a^ | 0.4906±0.05825 ^a^ | 0.75584±0.07533 ^a^ | 0.89711±0.15608 ^a^ |
| GNA | 0.65731±0.13657 ^b^ | 0.03996±0.01234 ^a^ | 0.009±0.00015 ^a^ | 0.00822±0.00138 ^a^ | 0.00803±0.00034 ^a^ | 0.01905±0.00713 ^a^ | 0.06202±0.01236 ^a^ | 0.11279±0.00853 ^a^ | 0.15118±0.02799 ^a^ | 0.11588±0.00734 ^a^ |
| 4OHI3M | 0.04627±0.00118 ^ab^ | 0.00477±0.00322 ^a^ | N.D. | N.D. | N.D. | 0.12361±0.06393 ^b^ | 0.09759±0.0197 ^ab^ | 0.00558±0.00251 ^a^ | 0.02131±0.00315 ^a^ | 0.0401±0.00398 ^ab^ |
| I3M | 0.01696±0.00405 ^a^ | 1.48443±0.24893 ^cde^ | 1.0674±0.09792 ^bc^ | 0.78097±0.01839 ^b^ | 0.77962±0.2449 ^b^ | 1.63849±0.30727 ^cde^ | 1.76824±0.08932 ^de^ | 1.12287±0.19279 ^bcd^ | 1.8478±0.0795 ^e^ | 1.55892±0.06546 ^cde^ |
| 4MOI3M | 0.03976±0.00228 ^ab^ | 0.14954±0.02826 ^b^ | 0.05386±0.01478 ^ab^ | 0.05626±0.00882 ^ab^ | 0.06943±0.0118 ^ab^ | 0.14298±0.00287 ^ab^ | 0.12489±0.01861 ^ab^ | 0.38152±0.06194 ^c^ | 0.02126±0.00956 ^a^ | 0.02106±0.00253 ^a^ |
| GIT | 0.65523±0.05027 ^b^ | 0.97649±0.07558 ^c^ | 0.52132±0.00044 ^b^ | 0.28992±0.05399 ^a^ | 0.30289±0.02773 ^a^ | N.D. | N.D. | N.D. | N.D. | N.D. |
| 1MOI3M | N.D. | 0.81106±0.01925 ^d^ | 0.44519±0.04299 ^c^ | 0.40279±0.0342 ^bc^ | 0.28184±0.05685 ^ab^ | 0.24342±0.07855 ^a^ | 0.357±0.02285 ^abc^ | 0.37686±0.05997 ^abc^ | 0.93458±0.04225 ^d^ | 0.42415±0.06708 ^bc^ |

**Table S2b.2**

|  | Aliphatic GSL | Indole GSL | Total GSL |
| --- | --- | --- | --- |
| Seeds | 27.05174±6.65733 ^c^ | 0.10298±0.00751 ^a^ | 27.15472±6.66484 ^c^ |
| 7d | 12.35875±3.88039 ^b^ | 2.44979±0.29966 ^de^ | 14.80855±4.18005 ^b^ |
| 14d | 5.46983±0.93426 ^a^ | 1.56645±0.15569 ^bc^ | 7.03627±1.08995 ^a^ |
| 21d | 3.59861±0.28918 ^a^ | 1.24002±0.06141 ^b^ | 4.83863±0.35059 ^a^ |
| 28d | 2.2546±0.30135 ^a^ | 1.13088±0.31355 ^b^ | 3.38548±0.6149 ^a^ |
| 60d | 0.49968±0.01665 ^a^ | 2.1485±0.45262 ^d^ | 2.64818±0.46927 ^a^ |
| 90d | 0.64836±0.11798 ^a^ | 2.34771±0.15048 ^de^ | 2.99607±0.26846 ^a^ |
| 120d | 0.75587±0.08016 ^a^ | 1.88683±0.31721 ^cd^ | 2.6427±0.39737 ^a^ |
| 150d | 2.08301±0.27189 ^a^ | 2.82494±0.13446 ^e^ | 4.90795±0.40635 ^a^ |
| 180d | 1.93789±0.32865 ^a^ | 2.04335±0.13905 ^cd^ | 3.98124±0.4677 ^a^ |

**Table S2c** Glucosinolates accumulation data in different organs

**Table S2c.1**

|  | Main roots | Lateral roots | Mature stems | Middle stems | Fresh stems | Mature leaves | Fresh leaves | Buds | Flowers | Immature fruits |
| --- | --- | --- | --- | --- | --- | --- | --- | --- | --- | --- |
| PRO | 2.55843±0.39578 ^b^ | 5.3865±0.39834 ^c^ | 0.08353±0.00758 ^a^ | 0.18135±0.01155 ^a^ | 0.32423±0.00439 ^a^ | 0.04092±0.00325 ^a^ | 0.24888±0.01656 ^a^ | 6.62216±0.57477 ^d^ | 8.66547±0.23255 ^e^ | 7.71764±0.49886 ^e^ |
| EPI | 2.16812±0.36337 ^b^ | 4.22635±0.27286 v | 0.2548±0.02349 ^a^ | 0.50713±0.02411 ^a^ | 1.09828±0.05371 ^ab^ | 0.34867±0.05036 ^a^ | 2.21907±0.19362 ^b^ | 12.30902±0.98957 ^d^ | 13.16122±0.43509 ^d^ | 12.05835±0.74828 ^d^ |
| GNA | 2.69143±0.13082 ^d^ | 5.40178±0.18601 ^e^ | 0.02673±0.00163 ^a^ | 0.04327±0.00299 ^a^ | 0.06742±0.00818 ^a^ | 0.01573±0.00204 ^a^ | 0.08829±0.00056 ^a^ | 1.60901±0.16286 ^c^ | 0.2973±0.03139 ^ab^ | 0.47808±0.04937 ^b^ |
| 4OHI3M | 0.02314±0.00419 ^bc^ | 0.00873±0.00136 ^ab^ | N.D. | N.D. | 0.00315±0.00038 ^a^ | N.D. | N.D. | 0.01515±0.00314 ^abc^ | 0.01918±0.00121 ^abc^ | 0.03044±0.01021 ^c^ |
| GTL | N.D. | N.D. | N.D. | N.D. | N.D. | N.D. | N.D. | 0.01638±0.00448 ^a^ | 0.17877±0.05425 ^a^ | 0.23402±0.04953 ^a^ |
| I3M | 1.07242±0.12295 ^c^ | 0.93329±0.15734 ^c^ | 0.01818±0.003 a | 0.00268±0.00042 a | 0.00987±0.00053 a | N.D. | 0.00356±0.00264 a | 0.06001±0.01125 a | 0.38473±0.06944 ^b^ | 0.61464±0.07615 ^b^ |
| 4MOI3M | 0.07082±0.01334 ^b^ | 0.02313±0.00692 ^a^ | N.D | N.D | N.D | N.D | N.D | N.D | 0.01278±0.00147 ^a^ | 0.01301±0.00158 ^a^ |
| 1MOI3M | 1.69006±0.17132 ^a^ | 2.07345±0.62469 ^a^ | 0.00816±0.00199 ^b^ | 0.00192±0.00015 ^b^ | 0.00254±0.00034 ^b^ | 0.02365±0.00153 ^b^ | 0.03476±0.00131 ^b^ | N.D | 0.07117±0.01186 ^b^ | 0.20557±0.00284 ^b^ |

**Table S2c.2**

| Organs | Aliphatic GSL | Aromatic GSL | Indole GSL | Total GSL |
| --- | --- | --- | --- | --- |
| Main roots | 7.41797±0.88997 ^b^ | N.D. | 2.83874±0.3118 ^c^ | 10.25672±1.20177 ^b^ |
| Lateral roots | 15.01463±0.85721 ^c^ | N.D. | 3.03859±0.79031 ^c^ | 18.05323±1.64752 ^c^ |
| Mature stems | 0.36507±0.0327 ^a^ | N.D. | 0.02674±0.00499 ^a^ | 0.3918±0.03769 ^a^ |
| Middle stems | 0.73175±0.03865 ^a^ | N.D. | 0.00468±0.00057 ^a^ | 0.73642±0.03922 ^a^ |
| Fresh stems | 1.48993±0.06628 ^a^ | N.D. | 0.01556±0.00125 ^a^ | 1.50549±0.06753 ^a^ |
| Mature leaves | 0.40532±0.05565 ^a^ | N.D. | 0.02365±0.00153 ^a^ | 0.42897±0.05718 ^a^ |
| Fresh leaves | 2.55623±0.21074 ^a^ | N.D. | 0.03832±0.00395 ^a^ | 2.59455±0.21469 ^a^ |
| Buds | 20.54018±1.7272 ^c^ | 0.01638±0.00448 ^a^ | 0.07252±0.01439 ^a^ | 20.62908±1.74607 ^d^ |
| Flowers | 22.12399±0.69903 ^c^ | 0.17877±0.05425 ^b^ | 0.48786±0.08398 ^ab^ | 22.79062±0.83726 ^d^ |
| Immature fruits | 20.25408±1.29651 ^c^ | 0.23402±0.04953 ^b^ | 0.86366±0.09078 ^b^ | 21.35175±1.43682 ^d^ |

**Table S2d** Glucosinolates accumulation data for different treatments

**Table S2d.1** AgNO_3_ treatment

|  | 0h | 3h | 6h | 9h | 12h | 24h | 48h | 72h |
| --- | --- | --- | --- | --- | --- | --- | --- | --- |
| PRO | 0.01524±0.00276 ^a^ | 0.04596±0.00273 ^bc^ | 0.01822±0.00275 ^a^ | 0.04836±0.01015 ^c^ | 0.15272±0.00808 ^e^ | 0.02911±0.00509 ^ab^ | N.D. | 0.07202±0.00442 ^d^ |
| EPI | 0.02443±0.00429 ^a^ | 0.06806±0.00264 ^bc^ | 0.09423±0.00697 ^c^ | 0.15364±0.02584 ^d^ | 0.20363±0.0097 ^e^ | 0.07596±0.01354 ^bc^ | 0.03821±0.00345 ^ab^ | 0.2164±0.01062 ^e^ |
| GNA | 0.02725±0.00494 ^a^ | N.D. | 0.04785±0.00367 ^b^ | 0.01446±0.00783 ^a^ | N.D. | 0.01423±0.00091 ^a^ | 0.00901±0.00022 ^a^ | 0.01602±0.00187 ^a^ |
| 4OHI3M | 0.00755±0.00111 ^a^ | 0.0752±0.05874 ^a^ | 0.2763±0.03302 ^b^ | 0.10395±0.01403 ^a^ | 0.0638±0.00868 ^a^ | 0.0095±0.00172 ^a^ | N.D. | 0.09594±0.01915 ^a^ |
| I3M | 0.21756±0.0004 ^a^ | 1.83656±0.39263 ^b^ | 1.58324±0.27195 ^b^ | 0.78364±0.10767 ^a^ | 0.75659±0.0738 ^a^ | 0.73402±0.03831 ^a^ | 0.63175±0.04449 ^a^ | 0.48354±0.06843 ^a^ |
| 4MOI3M | 0.06464±0.00636 ^ab^ | 0.1693±0.04826 ^c^ | 0.13202±0.02644 ^bc^ | 0.07927±0.01723 ^ab^ | 0.05812±0.00835 ^ab^ | 0.11213±0.00262 ^abc^ | 0.03597±0.00213 ^a^ | 0.15843±0.02682 ^c^ |
| 1MOI3M | 0.00494±0.00099 ^a^ | 0.14478±0.00374 ^e^ | 0.10988±0.01822 ^de^ | 0.03757±0.00325 ^ab^ | 0.09617±0.00966 ^c^ | 0.13856±0.00733 ^e^ | 0.05868±0.00459 ^bc^ | 0.1254±0.01219 ^de^ |
|  | 0h | 3h | 6h | 9h | 12h | 24h | 48h | 72h |
| Aliphatic GSL | 0.06692±0.00461 ^ab^ | 0.11402±0.00504 ^bc^ | 0.1603±0.00963 ^c^ | 0.21646±0.04094 ^d^ | 0.35635±0.01777 ^f^ | 0.11929±0.01286 ^c^ | 0.04722±0.00252 ^a^ | 0.30444±0.01523 ^e^ |
| Indole GSL | 0.29469±0.0057 ^a^ | 2.22583±0.35657 ^c^ | 2.10145±0.30707 ^c^ | 1.00443±0.08398 ^b^ | 0.97467±0.09682 ^b^ | 0.99421±0.02792 ^b^ | 0.7264±0.02847 ^ab^ | 0.86331±0.10831 ^b^ |
| Total GSL | 0.36161±0.00884 ^a^ | 2.33985±0.36079 ^c^ | 2.26175±0.31361 ^c^ | 1.22089±0.09844 ^b^ | 1.33102±0.11071 ^b^ | 1.1135±0.04073 ^b^ | 0.77362±0.02765 ^ab^ | 1.16775±0.11581 ^b^ |

**Table S2d.2** MeJA treatment

|  | 0h | 3h | 6h | 9h | 12h | 24h | 48h | 72h |
| --- | --- | --- | --- | --- | --- | --- | --- | --- |
| PRO | 0.01524±0.00276 ^a^ | 0.06808±0.00427 ^cd^ | 0.09228±0.01983 ^d^ | 0.05154±0.00566 ^bc^ | 0.0944±0.0036 ^d^ | 0.04267±0.00745 ^abc^ | 0.0342±0.00406 ^ab^ | N.D. |
| EPI | 0.02443±0.00429 ^a^ | 0.12794±0.00752 ^b^ | 0.23025±0.01309 ^c^ | 0.03266±0.00298 ^a^ | 0.13604±0.00215 ^b^ | 0.17874±0.03659 ^b^ | 0.03316±0.00601 ^a^ | 0.01489±0.00257 ^a^ |
| GNA | 0.02725±0.00494 ^ab^ | 0.02759±0.00031 ^ab^ | 0.03778±0.00866 ^ab^ | 0.13294±0.01025 ^d^ | 0.0502±0.01089 ^b^ | 0.08872±0.01877 ^c^ | 0.02734±0.00395 ^ab^ | 0.01484±0.00184 ^a^ |
| 4OHI3M | 0.00755±0.00111 ^a^ | N.D. | 0.04301±0.00281 ^a^ | N.D. | 0.03023±0.0113 ^a^ | 0.04132±0.0069 ^a^ | 0.02582±0.00018 ^a^ | 0.03855±0.01118 ^a^ |
| I3M | 0.21756±0.0004 ^a^ | 1.35044±0.32681 ^bc^ | 3.01351±0.12908 ^d^ | 1.70804±0.04867 ^bc^ | 1.095±0.08733 ^ab^ | 2.24268±0.03913 ^cd^ | 1.68869±0.4474 ^bc^ | 1.00087±0.01854 ^ab^ |
| 4MOI3M | 0.06464±0.00636 ^a^ | 0.13278±0.02163 ^b^ | 0.20871±0.02195 ^c^ | 0.06303±0.00715 ^a^ | 0.11517±0.00024 ^ab^ | 0.15769±0.02095 ^bc^ | 0.11126±0.00983 ^ab^ | 0.06916±0.0001 ^a^ |
| 1MOI3M | 0.00494±0.00099 ^a^ | 0.10773±0.02567 ^c^ | 0.22909±0.00287 ^d^ | 0.04676±0.0121 ^ab^ | 0.07186±0.00746 ^bc^ | 0.18895±0.0079 ^d^ | 0.06402±0.01077 ^bc^ | N.D. |
|  | 0h | 3h | 6h | 9h | 12h | 24h | 48h | 72h |
| Aliphatic GSL | 0.06692±0.00461 ^ab^ | 0.22361±0.01164 ^c^ | 0.3603±0.03194 ^e^ | 0.21714±0.00564 ^c^ | 0.28064±0.00364 ^d^ | 0.31013±0.03632 ^de^ | 0.0947±0.01264 ^b^ | 0.02973±0.00342 ^a^ |
| Indole GSL | 0.29469±0.0057 ^a^ | 1.59094±0.26453 ^bc^ | 3.49432±0.10983 ^e^ | 1.81783±0.04803 ^c^ | 1.31225±0.05921 ^bc^ | 2.63064±0.02226 ^d^ | 1.88978±0.4596 ^c^ | 1.10858±0.01846 ^b^ |
| Total GSL | 0.36161±0.00884 ^a^ | 1.81455±0.25732 ^c^ | 3.85463±0.08334 ^e^ | 2.03497±0.05324 ^c^ | 1.59289±0.06111 ^bc^ | 2.94077±0.01481 ^d^ | 1.98449±0.44876 ^c^ | 1.1383±0.02109 ^b^ |

**Table S2d.3** YE treatment

|  | 0h | 3h | 6h | 9h | 12h | 24h | 48h | 72h |
| --- | --- | --- | --- | --- | --- | --- | --- | --- |
| PRO | 0.01524±0.00276 ^a^ | 0.07081±0.01503 ^cd^ | N.D. | 0.02412±0.00228 ^ab^ | 0.10641±0.00865 ^e^ | 0.0767±0.00996 ^d^ | 0.02538±0.00215 ^ab^ | 0.04825±0.00099 ^bc^ |
| EPI | 0.02443±0.00429 ^a^ | 0.14867±0.02623 ^c^ | 0.03081±0.00329 ^a^ | 0.03957±0.00199 ^a^ | 0.15776±0.01282 ^c^ | 0.15065±0.01541 ^c^ | 0.10834±0.00341 ^b^ | 0.12152±0.00247 ^bc^ |
| GNA | 0.02725±0.00494 ^a^ | 0.03196±0.00585 ^a^ | 0.04082±0.01018 ^a^ | 0.01581±0.00199 ^a^ | 0.04126±0.00592 ^a^ | 0.0857±0.01335 ^b^ | 0.04191±0.00857 ^a^ | 0.03377±0.00455 ^a^ |
| 4OHI3M | 0.00755±0.00111 ^a^ | N.D. | 0.00248±0.00054 ^a^ | 0.00113±0.00016 ^a^ | 0.01292±0.01554 ^a^ | 0.00379±0.00101 ^a^ | 0.00221±0.00046 ^a^ | 0.0022±0.00081 ^a^ |
| I3M | 0.21756±0.0004 ^a^ | 0.79922±0.12192 ^ab^ | 1.26379±0.35135 ^bc^ | 1.65467±0.33581 ^c^ | 0.70759±0.06554 ^ab^ | 0.92591±0.14526 ^ab^ | 1.00609±0.19321 ^bc^ | 0.86367±0.04477 ^ab^ |
| 4MOI3M | 0.06464±0.00636 ^a^ | 0.08887±0.00803 ^a^ | 0.13548±0.02965 ^ab^ | 0.10402±0.01744 ^ab^ | 0.10074±0.00192 ^a^ | 0.36314±0.05671 ^c^ | 0.1707±0.01321 ^b^ | 0.09175±0.00475 ^a^ |
| 1MOI3M | 0.00494±0.00099 ^a^ | 0.07641±0.01097 ^bc^ | 0.08867±0.02348 ^bc^ | 0.10446±0.02125 ^c^ | 0.0056±0.00048 ^a^ | 0.04251±0.00077 ^ab^ | 0.06868±0.01466 ^bc^ | 0.00642±0.00059 ^a^ |
|  | 0h | 3h | 6h | 9h | 12h | 24h | 48h | 72h |
| Aliphatic GSL | 0.06692±0.00461 ^a^ | 0.25144±0.02836 ^c^ | 0.07642±0.01528 ^a^ | 0.0795±0.0026 ^a^ | 0.30543±0.02543 ^d^ | 0.31304±0.02845 ^d^ | 0.17563±0.01317 ^b^ | 0.20354±0.00396 ^bc^ |
| Indole GSL | 0.29469±0.0057 ^a^ | 0.965±0.10003 ^bc^ | 1.49042±0.32842 ^cd^ | 1.86428±0.2615 ^d^ | 0.82254±0.07095 ^b^ | 1.33535±0.14155 ^bcd^ | 1.24767±0.22101 ^bc^ | 0.96405±0.04296 ^bc^ |
| Total GSL | 0.36161±0.00884 ^a^ | 1.21644±0.11449 ^b^ | 1.56684±0.31727 ^bc^ | 1.94378±0.26337 ^c^ | 1.12797±0.05505 ^b^ | 1.64839±0.11311 ^bc^ | 1.4233±0.20836 ^bc^ | 1.16759±0.04001 ^b^ |

**Table S2d.4** Low temperature treatment

|  | 0h | 3h | 6h | 9h | 12h | 24h | 48h | 72h |
| --- | --- | --- | --- | --- | --- | --- | --- | --- |
| PRO | 0.01524±0.00276 ^a^ | 0.05294±0.00633 ^b^ | 0.16809±0.00356 ^d^ | 0.03999±0.0044 ^b^ | 0.11058±0.00642 ^c^ | 0.10657±0.00258 ^c^ | 0.10056±0.00602 ^c^ | 0.16671±0.00477 ^d^ |
| EPI | 0.02443±0.00429 ^a^ | 0.07599±0.00637 ^b^ | 0.35897±0.01047 ^f^ | 0.07741±0.00728 ^b^ | 0.22917±0.01681 ^d^ | 0.25753±0.00667 ^d^ | 0.17458±0.02062 ^c^ | 0.30298±0.01369 ^e^ |
| GNA | 0.02725±0.00494 ^a^ | 0.01402±0.00172 ^a^ | 0.06293±0.01099 ^ab^ | 0.02671±0.00218 ^a^ | 0.06619±0.00415 ^ab^ | 0.16524±0.04174 ^c^ | 0.09985±0.02957 ^b^ | 0.07492±0.01119 ^ab^ |
| 4OHI3M | 0.00755±0.00111 ^a^ | 0.00697±0.00141 ^a^ | 0.01159±0.00262 ^ab^ | 0.02469±0.00791 ^b^ | 0.01119±0.00208 ^ab^ | 0.00353±0.00002 ^a^ | 0.00583±0.00054 ^a^ | N.D. |
| I3M | 0.21756±0.0004 ^a^ | 0.36717±0.06036 ^a^ | 1.14085±0.25477 ^a^ | 0.37582±0.18287 ^a^ | 0.27552±0.09302 ^a^ | 1.08871±0.1663 ^a^ | 0.59826±0.48521 ^a^ | 0.65098±0.08741 ^a^ |
| 4MOI3M | 0.06464±0.00636 ^a^ | 0.11198±0.02106 ^ab^ | 0.1253±0.01917 ^ab^ | 0.1734±0.01156 ^bc^ | 0.06017±0.016 ^a^ | 0.21636±0.02759 ^c^ | 0.12543±0.00513 ^ab^ | 0.06998±0.00625 ^a^ |
| 1MOI3M | 0.00494±0.00099 ^a^ | 0.11643±0.01653 ^d^ | 0.07565±0.00625 ^bcd^ | 0.03356±0.00789 ^ab^ | 0.05181±0.01313 ^abc^ | 0.08487±0.02197 ^bcd^ | 0.10349±0.00801 ^cd^ | 0.11472±0.02201 ^d^ |
|  | 0h | 3h | 6h | 9h | 12h | 24h | 48h | 72h |
| Aliphatic GSL | 0.06692±0.00461 ^a^ | 0.14295±0.01267 ^b^ | 0.58998±0.02178 ^e^ | 0.14411±0.01316 ^b^ | 0.40594±0.01898 ^c^ | 0.52934±0.03169 ^d^ | 0.37499±0.00207 ^c^ | 0.54461±0.02097 ^de^ |
| Indole GSL | 0.29469±0.0057 ^a^ | 0.573±0.08704 ^ab^ | 1.35338±0.22793 ^c^ | 0.60746±0.1208 ^ab^ | 0.39868±0.08785 ^ab^ | 1.39347±0.15266 ^c^ | 0.833±0.33873 ^b^ | 0.83568±0.0762 ^b^ |
| Total GSL | 0.36161±0.00884 ^a^ | 0.71595±0.09387 ^ab^ | 1.94337±0.24143 ^d^ | 0.75157±0.11633 ^ab^ | 0.80463±0.08483 ^ab^ | 1.92281±0.18387 ^d^ | 1.20799±0.33774 ^bc^ | 1.38029±0.0857 ^c^ |

**Table S2d.5** SA treatment

|  | 0h | 3h | 6h | 9h | 12h | 24h | 48h | 72h |
| --- | --- | --- | --- | --- | --- | --- | --- | --- |
| PRO | 0.01524±0.00276 ^a^ | 0.08232±0.00859 ^bc^ | 0.06794±0.01398 ^b^ | 0.09899±0.0009 ^c^ | 0.02446±0.00152 ^a^ | 0.02819±0.00117 ^a^ | 0.07685±0.01069 ^bc^ | 0.0386±0.00041 ^a^ |
| EPI | 0.02443±0.00429 ^a^ | 0.31629±0.01863 ^e^ | 0.11554±0.0249 ^c^ | 0.2099±0.00049 ^d^ | 0.08552±0.005 ^bc^ | 0.09366±0.00744 ^bc^ | 0.18043±0.02571 ^d^ | 0.05363±0.0041 ^ab^ |
| GNA | 0.02725±0.00494 ^a^ | 0.16413±0.02034 ^d^ | 0.06562±0.01397 ^abc^ | 0.08411±0.00689 ^bc^ | 0.06363±0.00602 ^abc^ | 0.0729±0.00945 ^abc^ | 0.10258±0.00055 ^c^ | 0.05009±0.00671 ^ab^ |
| 4OHI3M | 0.00755±0.00111 ^a^ | N.D. | N.D. | 0.02483±0.00371 ^b^ | 0.02615±0.00092 ^b^ | 0.01264±0.00167 ^a^ | 0.00781±0.00068 ^a^ | 0.0107±0.00067 ^a^ |
| I3M | 0.21756±0.0004 ^a^ | 0.33292±0.05811 ^a^ | 1.45575±0.18433 ^b^ | 1.50893±0.27561 ^b^ | 1.47488±0.23273 ^b^ | 0.74695±0.00941 ^a^ | 0.47582±0.05795 ^a^ | 0.48367±0.10029 ^a^ |
| 4MOI3M | 0.06464±0.00636 ^ab^ | 0.05178±0.0089 ^ab^ | 0.0925±0.01349 ^abc^ | 0.14454±0.02839 ^c^ | 0.11883±0.01232 ^bc^ | 0.06118±0.00339 ^ab^ | 0.05715±0.0037 ^ab^ | 0.03679±0.00835 ^a^ |
| 1MOI3M | 0.00494±0.00099 ^a^ | 0.03526±0.00029 ^ab^ | 0.07011±0.01055 ^b^ | 0.11206±0.01393 ^c^ | 0.06235±0.01403 ^b^ | 0.06839±0.00338 ^b^ | 0.04176±0.00549 ^b^ | 0.04223±0.00865 ^b^ |
|  | 0h | 3h | 6h | 9h | 12h | 24h | 48h | 72h |
| Aliphatic GSL | 0.06692±0.00461 ^a^ | 0.56274±0.04408 ^e^ | 0.2491±0.04577 ^c^ | 0.39301±0.0039 ^d^ | 0.17362±0.00736 ^bc^ | 0.19475±0.01434 ^bc^ | 0.35986±0.03599 ^d^ | 0.14232±0.00736 ^ab^ |
| Indole GSL | 0.29469±0.0057 ^a^ | 0.41996±0.03459 ^ab^ | 1.61836±0.14734 ^c^ | 1.79036±0.31662 ^c^ | 1.6426±0.29746 ^c^ | 0.88915±0.01262 ^b^ | 0.58255±0.06317 ^ab^ | 0.57339±0.07455 ^ab^ |
| Total GSL | 0.36161±0.00884 ^a^ | 0.9827±0.01052 ^b^ | 1.86747±0.18485 ^c^ | 2.18336±0.31687 ^c^ | 1.81622±0.29927 ^c^ | 1.08391±0.0174 ^b^ | 0.94241±0.09569 ^b^ | 0.71571±0.06734 ^ab^ |

**Table S2d.6** NaCl treatment

|  | 0h | 3h | 6h | 9h | 12h | 24h | 48h | 72h |
| --- | --- | --- | --- | --- | --- | --- | --- | --- |
| PRO | 0.01524±0.00276 ^a^ | 0.12188±0.01167 ^c^ | 0.1567±0.01686 ^cd^ | 0.02186±0.00147 ^a^ | 0.02277±0.00504 ^a^ | 0.04151±0.00194 ^a^ | 0.17201±0.00344 ^d^ | 0.08519±0.02075 ^b^ |
| EPI | 0.02443±0.00429 ^a^ | 0.27971±0.02995 ^c^ | 0.59532±0.06582 ^d^ | 0.1002±0.00574 ^ab^ | 0.06017±0.02354 ^a^ | 0.02675±0.00084 ^a^ | 0.35023±0.02493 ^c^ | 0.1692±0.00127 ^b^ |
| GNA | 0.02725±0.00494 ^a^ | 0.11417±0.01103 ^c^ | 0.03595±0.00707 ^ab^ | 0.10601±0.01863 ^c^ | 0.02984±0.00949 ^a^ | 0.05267±0.00033 ^ab^ | 0.1689±0.00441 ^d^ | 0.08505±0.03079 ^bc^ |
| 4OHI3M | 0.00755±0.00111 ^a^ | 0.04627±0.00221 ^bc^ | 0.04795±0.00606 ^bc^ | 0.06269±0.0198 ^c^ | 0.005±0.00123 ^a^ | 0.02589±0.00292 ^ab^ | 0.04315±0.0052 ^bc^ | 0.02916±0.01151 ^abc^ |
| I3M | 0.21756±0.0004 ^a^ | 1.89187±0.1605 ^c^ | 1.55874±0.15725 ^bc^ | 1.38512±0.04135 ^bc^ | 0.77268±0.45308 ^ab^ | 0.79045±0.14318 ^ab^ | 1.07692±0.10322 ^abc^ | 1.08146±0.26216 ^abc^ |
| 4MOI3M | 0.06464±0.00636 ^ab^ | 0.25076±0.0391 ^e^ | 0.14393±0.01897 ^cd^ | 0.16519±0.00297 ^d^ | 0.05085±0.02046 ^a^ | 0.07554±0.0077 ^abc^ | 0.14002±0.01908 ^bcd^ | 0.07852±0.02736 ^abc^ |
| 1MOI3M | 0.00494±0.00099 ^a^ | 0.09524±0.01123 ^c^ | 0.10041±0.01598 ^c^ | 0.0982±0.0017 ^c^ | 0.07239±0.04113 ^ab^ | 0.00509±0.00043 ^a^ | 0.17902±0.01851 ^d^ | 0.03721±0.01505 ^ab^ |
|  | 0h | 3h | 6h | 9h | 12h | 24h | 48h | 72h |
| Aliphatic GSL | 0.06692±0.00461 ^a^ | 0.51577±0.0503 ^d^ | 0.78797±0.08795 ^e^ | 0.22807±0.02392 ^bc^ | 0.11278±0.03151 ^ab^ | 0.12093±0.00304 ^ab^ | 0.69114±0.02318 ^e^ | 0.33945±0.03403 ^c^ |
| Indole GSL | 0.29469±0.0057 ^a^ | 2.28413±0.17884 ^e^ | 1.85102±0.13642 ^de^ | 1.7112±0.03768 ^cd^ | 0.85992±0.30381 ^b^ | 0.89697±0.15301 ^b^ | 1.43912±0.10325 ^bcd^ | 1.22634±0.20268 ^bc^ |
| Total GSL | 0.36161±0.00884 ^a^ | 2.7999±0.21031 ^f^ | 2.639±0.1537 ^ef^ | 1.93927±0.05961 ^cd^ | 0.9727±0.32041 ^b^ | 1.0179±0.15511 ^b^ | 2.13026±0.12642 ^de^ | 1.56579±0.22609 ^c^ |

**Table S2d.7** Mechanical damage treatment

|  | 0h | 3h | 6h | 9h | 12h | 24h | 48h | 72h |
| --- | --- | --- | --- | --- | --- | --- | --- | --- |
| PRO | 0.01524±0.00276 ^a^ | 0.07047±0.01194 ^b^ | 0.02394±0.00053 ^a^ | 0.0211±0.0002 ^a^ | 0.02304±0.00055 ^a^ | 0.00526±0.00016 ^a^ | 0.0185±0.00208 ^a^ | 0.01622±0.00317 ^a^ |
| EPI | 0.02443±0.00429 ^a^ | 0.09174±0.02117 ^b^ | 0.08018±0.00232 ^b^ | 0.13638±0.02047 ^c^ | 0.08254±0.00307 ^b^ | 0.01975±0.00028 ^a^ | 0.0157±0.00041 ^a^ | 0.03161±0.00466 ^a^ |
| GNA | 0.02725±0.00494 ^ab^ | 0.02995±0.00162 ^ab^ | 0.0384±0.00026 ^b^ | 0.03976±0.00212 ^b^ | 0.02273±0.00271 ^a^ | 0.03116±0.00414 ^ab^ | 0.03593±0.00361 ^b^ | 0.02987±0.00279 ^ab^ |
| 4OHI3M | 0.00755±0.00111 ^a^ | 0.01973±0.00289 ^b^ | 0.01412±0.0021 ^ab^ | 0.00754±0.00154 ^a^ | 0.01416±0.00231 ^ab^ | 0.03041±0.0045 ^c^ | 0.01038±0.00171 ^ab^ | 0.01361±0.00101 ^ab^ |
| I3M | 0.21756±0.0004 ^ab^ | 0.71161±0.07427 ^c^ | 0.3256±0.04202 ^b^ | 0.12506±0.0146 ^a^ | 0.25482±0.03471 ^ab^ | 0.80617±0.0555 ^c^ | 0.13932±0.02175 ^a^ | 0.22723±0.00459 ^ab^ |
| 4MOI3M | 0.06464±0.00636 ^c^ | 0.05755±0.01041 ^c^ | 0.0332±0.00469 ^ab^ | 0.0178±0.00152 ^a^ | 0.02507±0.00139 ^a^ | 0.04883±0.00485 ^bc^ | 0.01529±0.00033 ^a^ | 0.02113±0.00186 ^a^ |
| 1MOI3M | 0.00494±0.00099 ^a^ | 0.06067±0.01005 ^c^ | 0.03369±0.00374 ^b^ | 0.00325±0.00021 ^a^ | 0.03558±0.00373 ^b^ | 0.00494±0.00048 ^a^ | 0.00249±0.00024 ^a^ | 0.00129±0.00022 ^a^ |
|  | 0h | 3h | 6h | 9h | 12h | 24h | 48h | 72h |
| Aliphatic GSL | 0.06692±0.00461 ^a^ | 0.19216±0.02497 ^c^ | 0.14252±0.00183 ^b^ | 0.19724±0.02117 ^c^ | 0.12831±0.00448 ^b^ | 0.05616±0.00453 ^a^ | 0.07013±0.00566 ^a^ | 0.0777±0.00649 ^a^ |
| Indole GSL | 0.29469±0.0057 ^abc^ | 0.84956±0.09435 ^d^ | 0.40661±0.05161 ^c^ | 0.15364±0.01656 ^a^ | 0.32962±0.03855 ^bc^ | 0.89034±0.05549 ^d^ | 0.16748±0.02264 ^a^ | 0.26325±0.00533 ^ab^ |
| Total GSL | 0.36161±0.00884 ^ab^ | 1.04172±0.09695 ^d^ | 0.54913±0.05009 ^c^ | 0.35088±0.03694 ^ab^ | 0.45793±0.03668 ^bc^ | 0.9465±0.05999 ^d^ | 0.23761±0.02655 ^a^ | 0.34095±0.00868 ^ab^ |

**Table S2d.8** ABA treatment

|  | 0h | 3h | 6h | 9h | 12h | 24h | 48h | 72h |
| --- | --- | --- | --- | --- | --- | --- | --- | --- |
| PRO | 0.01524±0.00276 ^a^ | 0.20795±0.01039 ^d^ | 0.03543±0.00405 ^ab^ | 0.17849±0.03743 ^d^ | 0.02981±0.0036 ^ab^ | 0.10686±0.01461 ^c^ | 0.0647±0.01406 ^bc^ | 0.03558±0.00526 ^ab^ |
| EPI | 0.02443±0.00429 ^a^ | 0.23171±0.00664 ^c^ | 0.01496±0.00117 ^a^ | 0.32324±0.04478 ^d^ | 0.05898±0.00679 ^a^ | 0.14976±0.02317 ^b^ | 0.12761±0.01458 ^b^ | 0.05497±0.00686 ^a^ |
| GNA | 0.02725±0.00494 ^a^ | 0.06231±0.01301 ^ab^ | 0.0803±0.01506 ^b^ | 0.10496±0.02072 ^b^ | 0.05674±0.0054 ^ab^ | 0.09399±0.0065 ^b^ | 0.15605±0.0252 ^c^ | 0.07812±0.01295 ^b^ |
| 4OHI3M | 0.00755±0.00111 ^a^ | 0.03659±0.00348 ^c^ | 0.00933±0.00022 ^ab^ | 0.01834±0.00228 ^b^ | 0.01449±0.00002 ^ab^ | 0.05481±0.00486 ^d^ | 0.0685±0.00134 ^e^ | 0.02809±0.00187 ^c^ |
| UN5 | N.D. | 0.02151±0.00133 ^a^ | 0.02226±0.01398 ^a^ | 0.02891±0.00377 ^ab^ | 0.02439±0.00009 ^ab^ | 0.05032±0.00888 ^ab^ | 0.05363±0.00409 ^b^ | 0.03464±0.00237 ^ab^ |
| UN6 | 0.00422±0.00164 ^a^ | N.D. | 0.00727±0.00313 ^a^ | N.D. | N.D. | N.D. | 0.14119±0.0179 ^b^ | 0.02861±0.0036 ^a^ |
| I3M | 0.21756±0.0004 ^a^ | 0.96387±0.02852 ^b^ | 0.55542±0.05615 ^ab^ | 0.62382±0.01058 ^ab^ | 0.36589±0.05943 ^a^ | 2.66863±0.34715 ^c^ | 2.26959±0.09161 ^c^ | 0.62479±0.02762 ^ab^ |
| 4MOI3M | 0.06464±0.00636 ^b^ | 0.05468±0.00307 ^ab^ | 0.07398±0.01317 ^b^ | 0.02698±0.00743 ^a^ | 0.02336±0.00149 ^a^ | 0.10904±0.0075 ^c^ | 0.11484±0.00468 ^c^ | 0.04787±0.01038 ^ab^ |
| 1MOI3M | 0.00494±0.00099 ^a^ | 0.18415±0.02047 ^c^ | 0.00552±0.00084 ^a^ | 0.17098±0.02301 ^c^ | 0.09377±0.01977 ^b^ | 0.03807±0.00241 ^a^ | 0.10945±0.00203 ^b^ | 0.00456±0.00082 ^a^ |
|  | 0h | 3h | 6h | 9h | 12h | 24h | 48h | 72h |
| Aliphatic GSL | 0.06692±0.00461 ^a^ | 0.50198±0.01088 ^d^ | 0.13069±0.01619 ^b^ | 0.60668±0.04348 ^e^ | 0.14553±0.01139 ^b^ | 0.35061±0.03145 ^c^ | 0.34836±0.01191 ^c^ | 0.16868±0.01139 ^b^ |
| Indole GSL | 0.29469±0.0057 ^a^ | 1.23927±0.03927 ^d^ | 0.64425±0.0595 ^bc^ | 0.84012±0.02008 ^c^ | 0.49751±0.07668 ^ab^ | 2.87055±0.24276 ^f^ | 2.56238±0.06857 ^e^ | 0.70532±0.02552 ^bc^ |
| Total GSL | 0.36161±0.00884 ^a^ | 1.74125±0.04771 ^c^ | 0.77494±0.07318 ^b^ | 1.4468±0.05304 ^c^ | 0.64304±0.08235 ^ab^ | 3.22115±0.24787 ^e^ | 2.91074±0.06244 ^d^ | 0.87399±0.02776 ^b^ |
