# Supplementary material for: Insights into glucosinolate accumulation and metabolic pathways in Isatis indigotica Fort
Source: BMC Plant Biol. 2022 Feb 22;22:78. doi: 10.1186/s12870-022-03455-6 (PMC8862337; doi:10.1186/s12870-022-03455-6)
Supplement: Supplementary file 18 — Additional file 18: Table S8. Pearson association analysis between the gene expression levels and GSL contents. The significant correlation is marked with an asterisk behind the number. [file 12870_2022_3455_MOESM18_ESM.docx]

**Table S8** Pearson association analysis between the gene expression levels and GSL contents

The significant correlation is marked with an asterisk behind the number.

**Table S8a** Results in different organs

| Different Organs | Organs | GSL-OH-1 | GSL-OH-2 | GSL-OH-3 | GSL-OH-like-1 | GSL-OH-like-2 |
| --- | --- | --- | --- | --- | --- | --- |
| PRO | correlation coefficient | -0.204 | -0.03 | 0.257 | 0.601 | -0.534 |
|  | significance | 0.599 | 0.94 | 0.504 | 0.087 | 0.138 |
| EPI | correlation coefficient | -0.002 | -0.233 | 0.309 | 0.783* | -0.422 |
|  | significance | 0.995 | 0.546 | 0.419 | 0.013 | 0.258 |

Note: The significant correlation is marked with an asterisk behind the number.

**Table S8b** Results in different periods

| Different Periods | Periods | GSL-OH-1 | GSL-OH-2 | GSL-OH-3 | GSL-OH-like-1 | GSL-OH-like-2 |
| --- | --- | --- | --- | --- | --- | --- |
| PRO | correlation coefficient | 0.015 | 0.118 | 0.055 | 0.029 | 0.061 |
|  | significance | 0.975 | 0.8 | 0.907 | 0.95 | 0.897 |
| EPI | correlation coefficient | -0.249 | 0.133 | -0.378 | -0.27 | -0.159 |
|  | significance | 0.59 | 0.776 | 0.404 | 0.558 | 0.734 |

Note: The significant correlation is marked with an asterisk behind the number.
