# Supplementary material for: Insights into glucosinolate accumulation and metabolic pathways in Isatis indigotica Fort
Source: BMC Plant Biol. 2022 Feb 22;22:78. doi: 10.1186/s12870-022-03455-6 (PMC8862337; doi:10.1186/s12870-022-03455-6)
Supplement: Supplementary file 19 — Additional file 19: Table S9. Selection criteria for samples of ten organs. [file 12870_2022_3455_MOESM19_ESM.docx]

**Table S9** Criteria for the selection of ten organs

| Organs | Abbreviation | Criterion |
| --- | --- | --- |
| Main roots | MR | The robust and straight root of an individual plant |
| Lateral roots | LR | The roots developed from the main root |
| Mature leaves | ML | The fifth to eighth cauline leaves when counting from the lowest leaves to the top |
| Mature stems | MS | The stems where mature leaves grew |
| Fresh leaves | FLE | The third to sixth cauline leaves when counting from the lowest branch with flowers to the root |
| Middle stems | MIS | The stems where fresh leaves grew |
| Fresh stems | FS | The main stem where the second to sixth branch developed |
| Buds | BUD | The unopened flowers |
| Flowers | FLO | The blooming flowers |
| Immature fruits | FR | Immature silicles from the plant |
